# *De novo* transcriptome and lipidome analysis of *Desmodesmus abundans* under model flue gas reveals adaptive changes after ten years of acclimation to high CO_2_

**DOI:** 10.1371/journal.pone.0299780

**Published:** 2024-05-17

**Authors:** Shirley Mora-Godínez, Carolina Senés-Guerrero, Adriana Pacheco

**Affiliations:** 1 Tecnologico de Monterrey, Escuela de Ingenieria y Ciencias, Monterrey, Nuevo Leon, Mexico; 2 Tecnologico de Monterrey, Escuela de Ingenieria y Ciencias, Zapopan, Jalisco, Mexico; Central University of Kerala, INDIA

## Abstract

Microalgae’s ability to mitigate flue gas is an attractive technology that can valorize gas components through biomass conversion. However, tolerance and growth must be ideal; therefore, acclimation strategies are suggested. Here, we compared the transcriptome and lipidome of *Desmodesmus abundans* strains acclimated to high CO_2_ (HCA) and low CO_2_ (LCA) under continuous supply of model flue gas (MFG) and incomplete culture medium (BG11-N-S). Initial growth and nitrogen consumption from MFG were superior in strain HCA, reaching maximum productivity a day before strain LCA. However, similar productivities were attained at the end of the run, probably because maximum photobioreactor capacity was reached. RNA-seq analysis during exponential growth resulted in 16,435 up-regulated and 4,219 down-regulated contigs in strain HCA compared to LCA. Most differentially expressed genes (DEGs) were related to nucleotides, amino acids, C fixation, central carbon metabolism, and proton pumps. In all pathways, a higher number of up-regulated contigs with a greater magnitude of change were observed in strain HCA. Also, cellular component GO terms of chloroplast and photosystems, N transporters, and secondary metabolic pathways of interest, such as starch and triacylglycerols (TG), exhibited this pattern. RT-qPCR confirmed N transporters expression. Lipidome analysis showed increased glycerophospholipids in strain HCA, while LCA exhibited glycerolipids. Cell structure and biomass composition also revealed strains differences. HCA possessed a thicker cell wall and presented a higher content of pigments, while LCA accumulated starch and lipids, validating transcriptome and lipidome data. Overall, results showed significant differences between strains, where characteristic features of adaptation and tolerance to high CO_2_ might be related to the capacity to maintain a higher flux of internal C, regulate intracellular acidification, active N transporters, and synthesis of essential macromolecules for photosynthetic growth.

## Introduction

Fossil fuel combustion is the major contributor to atmospheric CO_2_, followed by the cement industry, which accounts for 5% of global CO_2_ emissions and contributes to NO_x_ and SO_x_ (2.5 and 1.0% globally, respectively) [1–3]. Due to the accelerated increase in greenhouse gases, implementing mitigation strategies and energy alternatives is a global necessity. In particular, mitigation using microalgae represents an attractive strategy since they are considered robust photosynthetic microorganisms that can directly use CO_2_ from industrial combustion gases and possess CO_2_ fixation rates twelve times or higher than terrestrial crops [4–6].

Microalgae have been reported to use flue gas as a nutrient source and valorize gas components through biomass conversion into value-added compounds such as pigments, proteins, lipids, carbohydrates, and others [7–9]. Despite higher productivities in microalgae and accelerated growth under high concentrations of CO_2_, large-scale production is still a challenge. Therefore, elucidating microalgae metabolic mechanisms in response to high CO_2_ and other flue gas components would allow for establishing economically feasible and sustainable systems through strain acclimation and genetic manipulation to improve CO_2_ capture and the generation of metabolites of interest [10].

Transcriptomic analysis of microalgae under growth conditions of interest, such as high CO_2_, provides valuable information to understand and describe target pathways, even when the organism’s genome is unknown [11]. Thus, environmental microorganisms can be quickly evaluated for remediation processes and byproduct generation [12]. Most transcriptome studies have focused on the effect of growth conditions, such as high CO_2_ or limited nutrients (N, P, S), on lipid metabolism [10, 12–17]. However, information on microalgae genomes is lacking, with only around 149 genomes reported out of an estimated one million species [18, 19]. In addition, genetic variation, even among phylogenetically close species, in microalgae is high, making gene identification a challenge.

Previously our group demonstrated that the environmental chlorophyte *Desmodesmus abundans* tolerated, grew, and used components from cement flue gas as a nutrient source in a supply scheme of 24 h cycles (24 h of flue gas: 24 h without flue gas) [20]. Our main objective now is to assess the performance of two strains of *D*. *abundans* that have been acclimated for ten years to high (strain HCA) and low CO_2_ (strain LCA) when grown under a continuous gas supply and incomplete culture medium (BG11-N-S). The rationale is that the high CO_2_ strain possesses characteristics of interest for flue gas mitigation. We assembled and compared the strains’ *de novo* transcriptome and lipidome and characterized cell structure and biochemical composition to elucidate adaptive changes in the high CO_2_ strain.

## Materials and methods

### Microalgae and high CO_2_ acclimation strategy

**Fig 1:**
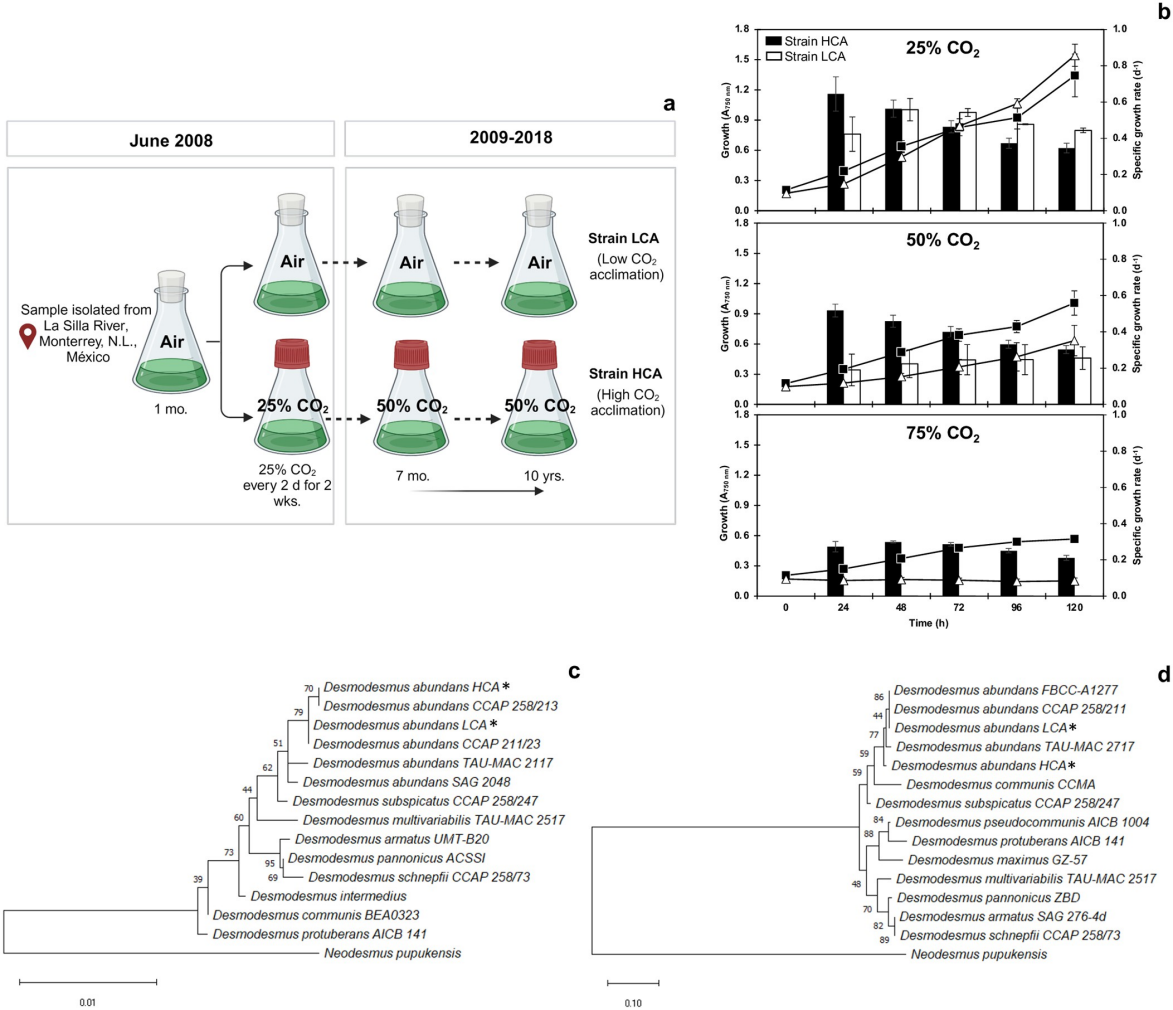
Schematic diagram of acclimation strategy to high CO_2_ (a), strain’s tolerance to high CO_2_ (b), and phylogenetic trees of the 18S rRNA gene (c) and ITS1-5.8S-ITS2 rRNA region (d) of *D*. *abundans* after 10 years of acclimation to low CO_2_ (strain LCA) and high CO_2_ (strain HCA). Phylogenetic trees as cladograms were constructed using the Neighbor-Joining method and *Neodesmus pupukensis* as the outgroup. Numbers represent the percentage of the bootstrap value of 1000 replicates, and asterisks indicate the studied strains.

*Desmodesmus abundans* strain RSM (UTEX 2976) was isolated from the environment in 2008 by our research group from a freshwater river (pH 7.4) in the city of Monterrey, Nuevo Leon, Mexico [20]. The strain was isolated after sample enrichment under air for one month and, subsequently, acclimated to an atmosphere of 25% v/v air/CO_2_ (Fig 1a). After six months, the 25% CO_2_ culture was exposed to an atmosphere of 50% CO_2_. All cultures were maintained for 10 years under these different atmospheres. In this study, the air strain was denominated low CO_2_ acclimated (LCA), and the 50% CO_2_ strain, high CO_2_ acclimated (HCA). Batch cultures were transferred every 7–10 days using 125 mL flasks containing 20 mL of BG11 medium, pH 7.4 [21]. The pH was not controlled during growth. Enriched CO_2_ atmospheres were obtained by using rubber stoppers, removing with a 60 mL syringe the corresponding volume of air from the headspace, and replacing it with 99.9% v/v CO_2_ (AOC Mexico, NL, Mexico). Cultures were incubated at 25 ± 2 °C, 60–70 μmol PAR photons m^-2^ s^-1^ of continuous light, and 110 rpm agitation in an orbital shaker.

### Microalgae growth under model flue gas

#### Cement model flue gas and cement kiln dust

Simulated gas components represented exhaust gases from a modern cement plant with a desulfurization system; concentrations used approximate maximum reported values [2, 22]. Hence, the model flue gas (MFG) comprised 250 000 ppm CO_2_, 700 ppm NO, and 100 ppm SO_2_ (in v/v). Independent mass flow meters were used to aerate each gas to a principal line, balanced with dry air, and delivered to the photobioreactor at a flow rate of 49 mL min^-1^ (0.05 vvm). Two Smart-Trak 2 Series 100 (Sierra Instruments, Monterrey, CA, USA) were used for CO_2_ and extra dry air, and two Side-Trak Series 840 for NO and SO_2_. The gas mixture was filtered at the reactor inlet with 0.2 μm PTFE membranes (Corning, NY, USA). CO_2_ (99.9%) and extra dry air were purchased from AOC Mexico (NL, Mexico), and 1.29% v/v NO (balance with N_2_) and 0.352% v/v SO_2_ (balance with N_2_) were provided from Praxair Mexico (NL, Mexico).

Cement kiln dust (CKD) was also considered a flue gas component and was used to control culture pH [6, 20]. CKD was collected from a local cement plant in Hidalgo (NL, Mexico) and sterilized by thinly spreading 150 mg into a polystyrene weighing dish and exposing it to UV light for 30 min under a laminar flow cabinet. CKD was delivered to the bioreactor (150 ppm) daily by dissolving 150 mg in 11 mL of fresh culture media.

#### Microalgae inoculum

The inoculum was previously grown in 500 mL flasks containing 75 mL of BG11 medium and 25 mL of culture (¾ log phase) for a final work volume of 100 mL, and 50% CO_2_ or air was supplied according to the strain used. At day 4 (¾ log phase), cultures were harvested by centrifugation (5000 rpm, 15 min, 4°C), and the pellet was washed with the experimental medium (BG11-N-S). Then, the inoculum was resuspended in 400 mL of BG11-N-S and added to the photobioreactor. Bioreactor initial biomass was normalized to Abs_750 nm_ 0.13 ± 0.02 (0.15 ± 0.05 g d.w. L^-1^).

#### Experimental conditions

**Fig 2:**
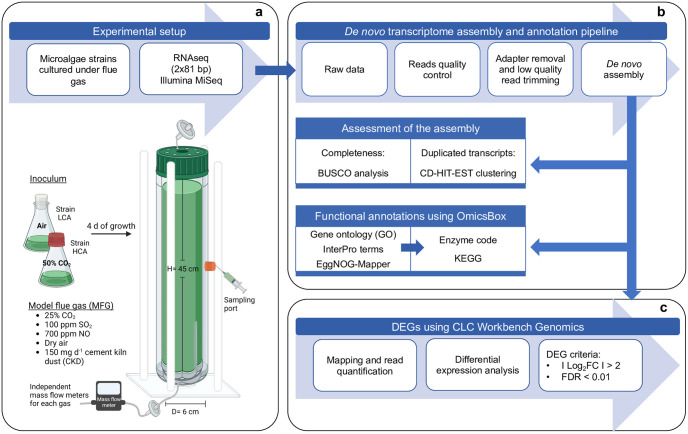
Experimental photobioreactor setup (a), *de novo* transcriptome assembly and annotation pipeline (b), and differentially expressed genes analysis (c) of *D*. *abundans* strains.

*D*. *abundans* strains were cultured in a 1 L customized column photobioreactor (6 cm diameter, 45 cm height) with 980 mL working volume at 25 ± 2 °C and continuous illumination (Fig 2a). The temperature was controlled by a glass water jacket covering the column. Four external fluorescent lamps (4000 K cool white, Philips) placed around the column provided 80–90 μmol PAR-photons m^-2^ s^-1^ to the vessel’s interior. The gas was supplied at a continuous flow rate of 49 mL min^-1^ (0.05 vvm) by a sparger stone at the bottom of the column. Metal meshes (1 mm) distributed at 5 cm along the column and covering the diameter of the bioreactor were used for gas retention.

As mentioned above, culture medium without nitrogen and sulfur (BG11-N-S) was used to evaluate the potential of MFG as a nutrient source. Therefore, NaNO_3_ and MgSO_4_·6H_2_O were replaced with NaCl and MgCl_2_.6H_2_O to provide Mg and Na in the same concentration and preserve solution osmolarity. Before column inoculation, 580 mL of BG11-N-S with 150 mg of CKD were aerated with MFG for 4 h to avoid microalgae starvation. During this period, 25% CO_2_ and a third of total NO and SO_2_ concentrations (i.e., 233 ppm NO and 40 ppm SO_2_) were supplied to the column. After inoculation, an acclimation period of 24 h was conducted under the same flue gas conditions. Finally, gas concentrations were set to the simulated MFG (25% CO_2_, 700 ppm NO, 100 ppm SO_2_), and 150 ppm w/v of CKD was added every 24 h. Cultures were sampled every 24 h by removing 11 mL of the culture and centrifuged (5000 rpm, 5 min, 4°C) to generate a cell pellet to determine biomass productivity. The supernatant was collected for chemical analysis.

### Growth determination

Culture growth was assessed daily during the experimental period by direct cell count under the microscope using a Neubauer hematocytometer. Growth kinetics were determined according to the model Ln(*X*/*Xo*) vs. time, where the linear portion of the curve represents exponential growth and the slope the specific growth rate. *X* is the daily biomass, and *Xo is* the initial biomass. Biomass productivity (g L^-1^ d^-1^) based on dry weight was measured daily by removing a sample (11 mL) that was centrifuged, the pellet stored at -80 °C before lyophilisation for 48 h and quantified by weight.

### Determination of pH, dO_2_, and dCO_2_

Culture pH, dissolved O_2_ (dO_2_), and dissolved CO_2_ (dCO_2_) were determined daily with 2 mL samples from the bioreactor using a pH sensor (Model Z001023551, AppliSens^®^, Scheidam, Holland), dO_2_ sensor (Model Z010023525, AppliSens^®^, Scheidam, Holland) and CO_2_ gas-sensing electrode (Van London-pHoenix Co., TX, USA). For dCO_2_ measurements, a 2 mL sample was added to a 15 mL Falcon tube containing 200 μL of ionic strength adjuster solution (Cat. No.C02IS01, London-pHoenix Co., TX, USA) to lower the pH to 4.8–5.2 and convert all carbonate species (bicarbonate and carbonate) to gaseous CO_2_. Readings were taken once the electrodes were stabilized. The CO_2_ sensor was calibrated by bubbling different CO_2_/air (v/v) concentrations into deionized water at 25 °C; once the signal stabilized (around 1–5 min, no more than 20 min), the corresponding electrode potential (mV) was recorded. A five-point calibration curve was performed in a range of 0.04 to 40% v/v CO_2_/air (y = (1.949*x) − 95.406, R^2^ = 0.964), as recommended by the provider when working above the limit of detection of the electrode (i.e., 1x10^-2^ M or 440 ppm). The calibration of the O_2_ sensor was performed by bubbling air into deionized water until saturation at 25 °C.

### Chemical analysis of NO_2_^−^, NO_3_^−^ and SO_4_^2-^ in solution

Nitrite, nitrate, and sulfate in solution were monitored daily following standard methods for water and wastewater analysis [23] 4500-NO_2_− B Colorimetric Method, 4500-NO_3_− B Ultraviolet Spectrophotometric Screening Method, and 4500- SO_4_^2-^ E Turbidimetric Method, respectively.

### Phylogenetic analysis

#### DNA extraction and PCR amplification

Exponential growth cultures (day 4) were harvested by centrifugation, and the fresh pellet was stored at -80 °C. DNA extraction (100 mg fresh weight) was performed using the FastDNA^™^ Spin Kit for Soil (MP Biomedicals, CA, USA). The quantity and quality of the DNA were analyzed using a NanoDrop^*™*^ 1000 Spectrophotometer (ThermoFisher Scientific, MA, USA). The 18S rDNA region and the ITS1-5.8S-ITS2 rDNA region were PCR amplified using a T100^™^ Thermal Cycler (BIO-RAD, CA, USA). 18S rDNA primers (18S-forward: 5’- TTTCTGCCCTATCAACTTTCGATG-3’, 18S-reverse: 5’- TACAAAGGGCAGGGACGTAAT-3’) generated a fragment size of c. 1200 bp and ITS primers (ITS-forward: 5’- TTCCTCCGCTTATTGATATGC-3’, ITS-reverse: 5’- ACCTAGAGGAAGGAGAAGTCGTAA-3’) an amplification product of c. 650 bp [24, 25]. For both genes, PCR reactions consisted of 5 μL of GoTaq^®^ Green Master Mix 2x (Promega, WI, USA), 2.5 μL of each primer, 100 ng DNA, and water to a final volume of 25 μL. The PCR protocol for both regions contemplated an initial denaturation step of 96 °C for 4 min followed by 36 cycles of denaturation at 96 °C for 30 s, annealing at 50 °C for 30 s, and extension at 72 °C for 1 min, with a final extension step of 72 °C for 6 min. For ITS region amplification, the annealing temperature was changed to 48 °C for the same time. PCR fragment size was verified in 1.5% w/v agarose gel.

#### Cloning and sequencing

PCR products were cloned using the Zero Blunt^®^ TOPO^®^ PCR Cloning Kit (Life Technologies, CA, USA) and the pCR^™^II-Blunt-TOPO^®^ vector. Ligation volumes were modified to 1 μL PCR product, 0.3 μL saline solution, 0.3 μL plasmid, and 0.3 μL water. To confirm clones, direct colony PCR was performed. Reactions contained 5 μl GoTaq^®^ Green Master Mix 2x (Promega, WI, USA), 0.1 μl of 10 uM primers (M13F and M13R), 4.8 μl water, and bacterial colony. PCR conditions contemplated an initial denaturation set of 94 °C for 10 min to lysate the cells, followed by 25 cycles of denaturation at 94 °C for 1 min, annealing at 55 °C for 1 min, extension at 72 °C for 1 min, and a final extension step of 72 °C for 7 min. Products were verified in 1.5% w/v agarose gel. After, selected colonies were grown in 2 mL of Luria-Bertani (LB) medium (Sigma-Aldrich, MO, USA) containing kanamycin (Sigma-Aldrich, MO, USA) and incubated in a shaker overnight at 37 °C. Then, plasmids were purified using the PureYield^™^ Plasmid Miniprep System (Promega, WI, USA). Samples were sequenced at the Langebio Genomic Services Laboratory (GTO, Mexico).

#### Phylogenetic analysis

First, a consensus sequence of each region of interest was generated using the forward and reverse sequences and the program SeqAssem ver.07/2008. This sequence was analyzed using the Basic Local Alignment Search Tool (BLAST) of the National Center for Biotechnology Information (NCBI) database to identify similar sequences. Twelve to thirteen sequences were obtained for each region, and a phylogenetic tree was constructed using the Neighbor-Joining method with 1000 bootstrap replicates and the Jukes-Cantor model using MEGA11 [26].

### RNA-seq analysis

#### RNA extraction

Samples from the photobioreactor at exponential growth (day 4) were centrifuged (5000 rpm, 5 min, 4 °C), the supernatant discarded, and the cell pellet frozen in liquid nitrogen and stored at -80 °C until processing. Cell rupture (60–80 mg frozen pellet) was performed using lysis matrix E (MP Biomedicals, CA, USA) and 1 mL of lysing buffer (RNeasy Plant Mini Kit; Qiagen, USA) in the FastPrep^®^-24 Homogenizer (MP Biomedicals, CA, USA) at 6 m s^-1^ for 40 s. The mixture was centrifugated at 12000 rpm for 10 min, and the supernatant was used for RNA purification. The following steps were performed as indicated in the RNeasy Plant Mini Kit (Qiagen, USA) protocol. RNA quality and quantity were evaluated by agarose gel electrophoresis and using the Nanodrop 1000 spectrophotometer (Thermo Scientific, USA) and the Agilent 2100 Bioanalyzer (Agilent, USA). Quality parameters were an rRNA ratio [28S/18S] ≥ 1, OD 260/280 ≥ 1.9, OD 260/230 ≥ 1.5, and RNA integrity number (RIN) > 7.

#### RNA-seq library construction

A total of six samples, three replicates for each strain, were used to generate cDNA libraries using 500 ng of total RNA and following the TruSeq RNA Sample Prep v2 LS Protocol (Illumina, CA, USA). Pooled libraries were normalized to 11 pM, and 1% v/v of 11 pM PhiX sequencing control was added. A paired-end sequencing run was performed (2x81 bp) using the MiSeq Reagent Kit v3 in the MiSeq Sequencer (Illumina, USA).

#### Transcriptome *de novo* assembly and functional annotations

A total of 8.19 Gb of information were used for *de novo* transcriptome assembly, which included 2.75 Gb of a previous run (2x306 bp) to support the assembly. The transcriptome pipeline of analysis is shown in Fig 2b and 2c. Data processing and assembly were performed using CLC Workbench Genomics 11.0 (Qiagen, USA). Sequencing QC reports were generated to assess raw data quality. Adaptors and low-quality reads were removed using the Trim Reads tool; trimmed reads were merged and used for *de novo* assembly. The parameters for assembly were a word size of 24, a bubbled size of 50, a penalty value of 3 for mismatch, deletion, and insertion costs; 200 bp as minimum contig length, 0.5 for length fraction, and 0.95 of similarity fraction. Once assembled, the *de novo* transcriptome was evaluated according to the read percentage mapping back to the assembly and N50 length statistics from CLC Workbench Genomics. Completeness was assessed using the BUSCO program (Benchmarking Universal Single-Copy Orthologs) [27] against the eukaryotic lineage dataset (303 BUSCOs from 100 species), and redundant contigs were determined utilizing the CD-HIT-EST program [28] for sequence identities of 95 and 100%.

Gene ontology terms were assigned using OmicsBox 2.1.14 [29]. BLASTx was performed against the Viridiplantae, Cyanobacteria, Rhodophyta, and other phototrophic eukaryote subsets of the non-redundant protein sequences (nr v5), followed by the GO mapping and annotation steps. After, InterProScan and EggNOG-Mapper were runned, and GO terms were merged with the previous annotation. Enzyme code (EC) and biochemical pathways were assigned using the Kyoto Encyclopedia of Genes and Genomes (KEGG) [30].

#### Differential expression analysis

Differential expression analysis between strains consisted of 6 034 105, 4 960 949, and 5 496 127 reads for strain HCA replicates, and 4 898 045, 6 439 209, and 5 305 484 reads for LCA. Read quantification and differential analysis were performed using CLC Workbench Genomics 11.0 (Fig 2c). The trimmed paired reads from each strain were mapped against the *de novo* transcriptome and quantified using the RNA-seq Analysis tool with default parameters. Expression tracks obtained from the quantification step were used to identify differentially expressed genes (DEGs) between the HCA strain compared to the LCA strain. The RNA-seq Analysis tool fits a generalized linear model (GLM) using the Wald test, assumes a negative binomial distribution for the read count, and p-values were corrected by false discovery rate (FDR) using Benjamini and Hochberg’s method [31]. Threshold values were set as FDR<0.01 and log_2_(FC)>2.

### Real-time qPCR analysis

cDNA from previously extracted RNA was generated using the SuperScriptTM III Reverse Transcriptase Kit (Invitrogen, CA, USA). Transcriptome contigs encoding nitrate, ammonium, and urea transporters were selected for primer design (S1 Table). In addition, the 18S rDNA transcript was also considered as the housekeeping gene. Primer concentrations were optimized, and standardization curves were generated. The optimized concentrations of cDNA and primers are shown in S1 File. qPCR was performed using the 7500 Fast Real Time PCR system (Applied Biosystems, USA). Reactions in triplicate for each sample contained 5 μL of Brilliant III Ultra-Fast SYBR^®^ Green qPCR Master Mix (Agilent Technologies, CA, USA), 0.3 μL of each primer, 2.4 μL of water, and 2 uL of cDNA. The qPCR conditions were 95 °C for 3 min followed by 40 cycles of 95 °C for 20 s, 60 °C for 15 s, and 72 °C for 30 s. The dissociation curve conditions were 72 °C for 30 s, a ramp of 2.2% to 95 °C, and 95 °C for 30 s. Relative expression was analyzed by the 2^-ΔΔCT^ method [32].

### Lipidome analysis

#### Lipid extraction

Lyophilized biomass from day 4 and 5 was processed to generate a lipid extract following the protocol reported by [33]. Briefly, in a 15 mL polypropylene tube, 5 mg of dried biomass was added with 1.5 mL 100% methanol (BDH, PA, USA), 500 μL HPLC water, and 10 glass beads (1 mm diameter). Cell lysis was performed using a FastPrep^®^-24 Homogenizer (MP Biomedicals, CA, USA) at 6 m s^-1^ for 40 s. Lysed cells protected from light were mixed in a vortex, sonicated for 1 min, and left at room temperature for 1 min, and the process was repeated five times. Then, the content was transferred into a glass tube, and 5 mL methyl tert-butyl ether (J.T. Baker, PA, USA) was added, vortexed, sonicated for 1 min, and incubated at room temperature in an orbital shaker at 250 rpm for 1 h. After, 1.25 mL HPLC water was added, and the mixture vortexed, sonicated for 1 min, and incubated as previously described for 10 min. Samples were centrifuged (500 g, 20 min), and the organic phase (upper) was recovered in a glass tube. Then, 2 mL of the upper phase of a solution of methyl tert-butyl ether:methanol:water (20:6:7), prepared the previous day, was used to wash the remaining aqueous phase as described above, and the organic phase recovered and combined with the first. Then, the organic phase was evaporated at 55 °C using a CentriVap Concentrator (LABCONCO, MO, USA), and the lipid extract was stored at -80 °C.

#### Chromatographic separation

The lipid extracts were resuspended in 500 μL 100% isopropanol (BDH, PA, USA), centrifuged (10 000 g, 1 min), and 400 μL of the supernatant recovered. Samples were separated by HPLC (Series 1100; Agilent, CA, USA) coupled via ESI to a TOF MS Detector (G1969A; Agilent, CA, USA) system, using a Luna C18(2) column (150x2 mm, 3 μm; Phenomenex, CA, USA). The gradient elution program consisted of water:acetonitrile (4:1 v/v; phase A) and isopropanol:acetonitrile (9:1 v/v; phase B) as mobile phases, both modified with 10 mM ammonium acetate and 0.1% formic acid. The program for sample separation was set at 55 °C, and the elution gradient had a constant flow of 0.2 mL min^-1^. A linear gradient was generated with ramps from 40 to 43% B (6 min), changing immediately to 50% B and increasing linearly to 54% B until minute 36; then jumped to 70% B and increased to reach 99% B by minute 54, at this point the column returned to the initial condition (40% B), where it equilibrated (10 min). ESI drying gas, nitrogen, was set to 13 L h^-1^, at 350 °C, using a nebulizer pressure of 35 psig, with a capillary voltage of 4.5 kV to favor fragmentation. The optical parameters were set to 250 V for the octupole radio frequency voltage (Oct RFV), 225 V for the fragmentor, and 60 V for the skimmer.

Runs were performed to acquire mass spectra in positive mode, and files were saved in profile mode with an m/z range from 150 to 1500. Readings were obtained at 0.94 cycles per second, with 10,000 transients per scan. Samples were injected randomly, beginning with three ‘dummy’ runs and three QC equidistant runs that consisted of a mix of all samples in the same amount.

#### Feature detection

Raw files were converted to CDF with Agilent’s Translator Utility (Agilent, CA, USA) and processed with MZmine 2.28 platform [34]. Peak detection was performed using the GridMass algorithm [35] with the following parameters: minimum height of 30 000 counts, m/z tolerance of 0.05, retention time (RT) window between 0.1 and 1 min, smoothing time of 0.1 min, intensity similarity ratio of 0.5, and smoothing m/z of 0.05. Isotopes were grouped using an m/z tolerance of 0.001 or 10 ppm, RT tolerance of 0.25, and maximum charge of 2, and a monotopic shape was assumed. The feature alignment was done using the RANSAC algorithm, using an m/z tolerance of 0.025 (50 ppm), an RT tolerance of 1 min before and 0.8 after RT correction, a minimum of 20% points matching the non-linear model below a threshold of 0.4 min. Peaks were filtered using 2 as the minimum of peaks in a row. Gap filling was performed using the same RT and gap range m/z filler algorithm with an m/z tolerance of 0.025 (50 ppm).

#### Assignation of identity

Putative feature identities were assigned using the Lipid MAPS^®^ REST service, as previously performed by [33, 36]. The generated m/z minus the adduct mass and an m/z tolerance of 0.05 were used for searching in the REST service. The molecular formula, name, and Lipid MAPS^®^ classification were retrieved. The most probable formula and class were assigned according to the highest frequency of the data retrieved.

#### Statistical analysis

All statistical analyses were performed using the R programming language. Feature areas were log-transformed and quantile-normalized. Comparison between strains’ lipidomes was performed using a t-test and *FDR-corrected p-values* [31]. An FDR value <0.05 was considered significant. Then, using the significant features, a cloud plot was created by plotting size and transparency in function of the log_2_ (FC) and FDR *p-value*, respectively.

### Cell morphology and structural analysis

Cultures were analyzed at early stationary phase (day 5) using scanning electron microscopy (SEM) and transmission electron microscopy (TEM). A sample of 2 mL was centrifuged (2250 rpm, 10 min, 20 °C), the pellet washed with 300 μL of fresh culture medium, fixed with 2.5% w/v glutaraldehyde Grade I (Sigma-Aldrich, MO, USA) solution in 0.1 M phosphate buffer for 24 h, and washed 3X with 300 μL of 0.1 M phosphate buffer with an incubation period of 3 min. For SEM, fixed cells were then washed 2X with 70% v/v ethanol (DEQ, México), placed in a conductive double side carbon tape, dried in a desiccator for 2 h, and coated with gold for charge dissipation. Images were obtained using a Zeiss EVO MA25 SEM (Zeiss, Germany) with an accelerating voltage of 20 kV. For TEM, fixed cells were stored at 4 °C and analyzed by the Institute of Cellular Physiology of the National Autonomous University of Mexico (CDMX, Mexico). Measurements of cell size and wall thickness from TEM images were analyzed using ImageJ 2.0.

### Pigment content, protein, and starch

Biomass composition was analyzed at early stationary phase (day 5). Pigments were quantified spectrophotometrically using the protocol described by Mora-Godínez *et al*. [33] In a 2 mL tube with 1.0–1.5 mg of lyophilized biomass, 2 mL of 100% v/v methanol (BHD, PA, USA) were added. The solution was sonicated for 1 min and incubated at 45 °C for 24 h. After, 250 μL of the solution were placed in a clear microplate and analyzed using a Synergy HT Microplate Reader (Bio Tek, Vermont, USA). Pigments were calculated using the following equations [37]: *Chl*_*a*_ (μg mg^-1^) = (16.72 (A_665nm_- A_750nm_)– 9.16 (A_652nm_-A_750nm_)) * (V*e*/(X*L)), *Chl*_*b*_ (μg mg^-1^) = (34.09 (A_652nm_ -A_750nm_)– 15.28 (A_665nm_ -A_750nm_)) * (V*e*/(X*L)), and carotenoids (μg mg^-1^) = ((1000 (A_470nm_ -A_750nm_)– 1.63 *Chl*_*a*_*−* 104.9 *Chl*_*b*_)/221) * (V*e*/(X*L)). Where, *Chl* corresponds to chlorophyll concentration, A denotes absorbance at a specific wavelength, Ve is the extract volume (mL), *X* is biomass (mg d.w.), and L is the cuvette light path (cm). Crude protein and starch content (50 mg of lyophilized biomass) were determined using the micro-Kjeldahl Official method AOAC 978.02. and the Starch Assay kit STA20 (Sigma-Aldrich, MO, USA), respectively.

## Results

### Tolerance to CO_2_ and phylogenetic analysis

Growth of strains under various high CO_2_ atmospheres was assessed as a first approach to validate the acclimation strategy (Fig 1a and 1b). In contrast to strain HCA, strain LCA was not able to grow at 75% CO_2_ and a 37% reduction in final Abs_750nm_ compared to strain HCA was observed at 50% CO_2_. Comparable growth was observed at 25% CO_2_. However, specific growth rates in all tested concentrations were lower at the beginning of the experimental period for strain LCA (Fig 1b).

To confirm the identity of the environmental strains under the different regimens of CO_2_ acclimation, sequences of the 18S rDNA region and ITS1-5.8S-ITS2 rDNA region were analyzed, and phylogenetic trees constructed (Fig 1c and 1d). Both strains 18S rDNA sequences showed a 100% similarity to other *D*. *abundans* species, particularly to strain CCAP 258/213 (GenBank: MK541740.1) and CCAP 211/23 (MG022724.1) for strain HCA and LCA, respectively (Fig 1c). Similarly, the ITS1-5.8S-ITS2 rDNA region analysis grouped the strains in the same clade of *D*. *abundans* species (Fig 1d). Both analyses located the strains in different branch positions because of nucleotide variations in the 18S rDNA (c. 1327 bp) and ITS sequences (c. 678 bp).

### Microalgae growth under model flue gas

**Fig 3:**
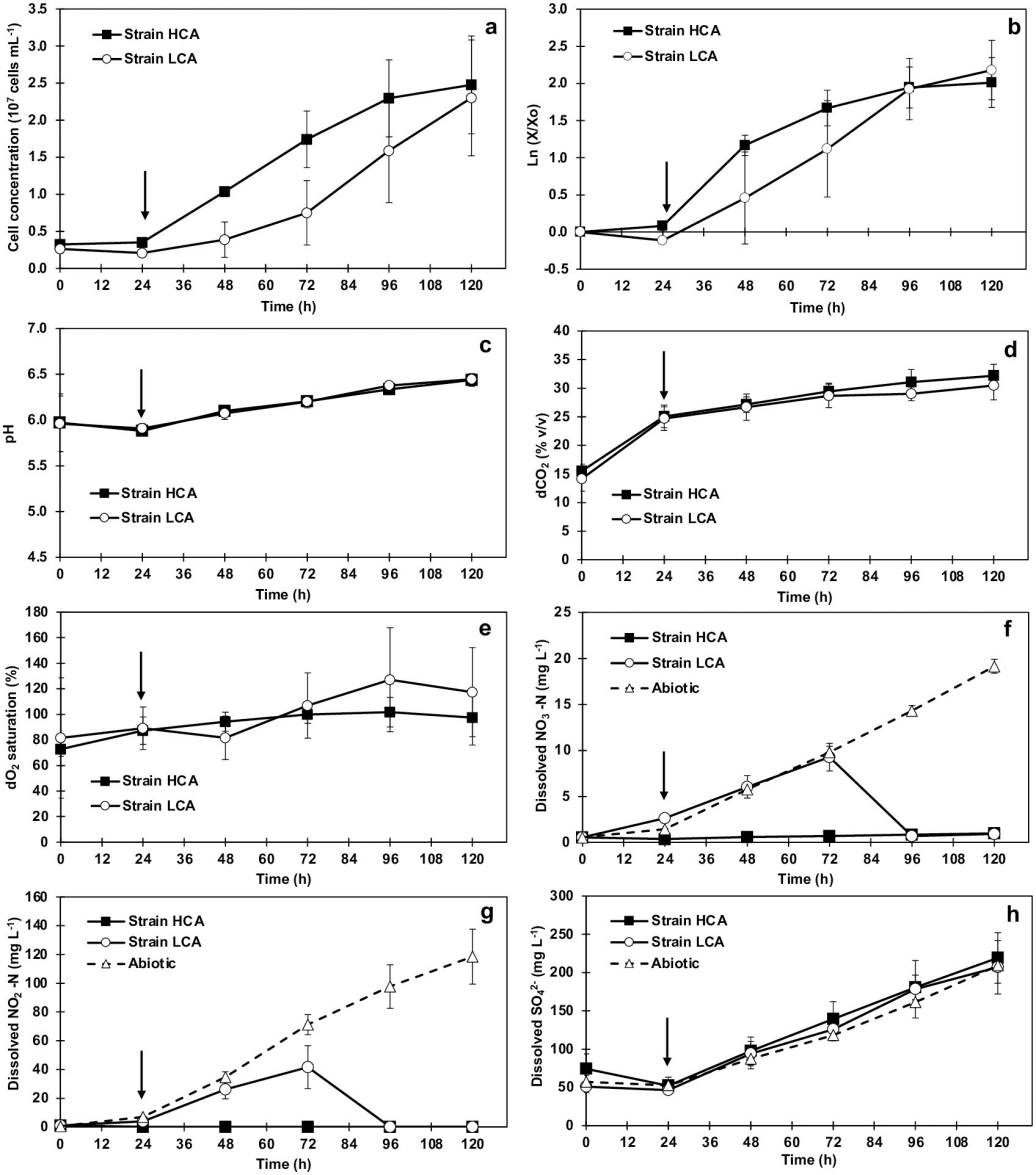
Arithmetic (a) and semi-log (b) growth curves, pH (c), dCO_2_ (d), dO_2_ saturation (e), dissolved NO3—N (f), dissolved NO_2_-N (g) and dissolved SO42- (h) of *D*. *abundans* strains under model flue gas in a 1 L column photobioreactor at 24 °C, 80–90 μmol PAR-photons m^-2^s^-1^ and 24 h light. Model flue gas consisted of (v/v): 250 000 ppm CO_2_, 700 ppm NO, and 100 ppm SO_2_. Values represent average ± SD (n = 3–6). Time 0 h corresponds to values after 4 h supply of a third NO and SO_2_, and 25% CO_2_, to avoid starvation in BG11-N-S medium. Arrows indicate addition of total amount of NO_x_ and SO_x_ after 24 h of stabilization of the system.

Growth was assessed daily in the designed photobioreactor (Fig 2a). After an adjustment period of 24 h, strain HCA grew at a higher rate and entered the stationary phase a day earlier than strain LCA (Fig 3a and 3b). However, both strains presented similar maximum specific growth rates of 0.79 ± 0.14 and 0.65 ± 0.13 d^-1^ for HCA and LCA, respectively (Tukey’ HSD test, *α* = 0.05) (Table 1). In congruence, both cultures achieved similar final cell concentrations (2.5 ± 0.7 and 2.3 ± 0.8 x 10^7^ cells mL^-1^), but HCA reached this biomass a day before (Fig 3a). Biomass productivities for both strains during exponential growth were in average 0.33 and 0.34 g L^-1^ d^-1^ and gas fixation rates were 0.61 and 0.62 g L^-1^ d^-1^ for CO_2_, 0.06 and 0.07 g L^-1^ d^-1^ for NO_x_, and, in both strains, 0.006 g L^-1^ d^-1^ for SO_x_.

**Table 1:** Growth parameters of *D*. *abundans* strains HCA and LCAunder model flue gas in BG-11-N-S medium in a 1 L column photobioreactor with continuous flow (0.05 vvm), 24 °C, 80–90 μmol PAR-photons m^-2^s^-1^ and 24 h light.

Strain	Maximum specific growth rate (d^-1^)	Productivity (g L^-1^ d^-1^)^a^	Fixation rate (g L^-1^ d^-1^)^b^		
			CO_2_	NO_x_	SO_x_
HCA	0.79 ± 0.14	0.33 ± 0.06	0.61 ± 0.11	0.064 ± 0.015	0.006 ± 0.001
LCA	0.65 ± 0.13	0.34 ± 0.13	0.62 ± 0.23	0.068 ± 0.026	0.006 ± 0.002

^a^During exponential growth in a dry weight basis.

^b^Based on biomass productivity during exponential growth accordingly to Jian *et al*. [38].

HCA: high CO_2_ acclimated. LCA: low CO_2_ acclimated. Model flue gas composition in dry air (v/v): 25% CO_2_, 700 ppm NO, 100 ppm SO_2,_ and 150 ppm (w/v) CKD were added every 24 h. Values represent average ± SD (n = 3–6). Statistical differences were not found between strains (t-test, *α* = 0.05).

Photobioreactor runs were monitored daily by measuring pH, dCO_2_, and dO_2_ (Fig 3c–3e), as well as dissolved NO_x_ and SO_x_ (Fig 3c–3h). After 4 h of aeration and before inoculation, the initial pH was 5.96 (Fig 3c). When the total amount of NO_x_ and SO_x_ was supplied at 24 h, the pH was not drastically affected and gradually increased to around 6.44. Similarly, dCO_2_ increased from around 25% v/v at 24 h to a maximum average of 31.3% after 5 d of continuous aeration (Fig 3d). dO_2_ saturation reached a maximum average of 107.4% (Fig 3e). After 4 h of bubbling NO gas, 0.6 mg L^-1^ NO_3_^−^-N and 0.5 mg L^-1^ NO_2_^−^-N were detectable at the beginning of the run (Fig 3f and 3g). These values reached 19 and 118 mg L^-1^ N in abiotic conditions after 5 d of aeration. In addition, differences between strains were evident in dissolved N consumption. In strain LCA, NO_3_^−^-N and NO_2_^−^-N accumulated to 9 ± 1 and 42 ± 5 mg L^-1^ N by day 3 and then decreased, while for HCA, only NO_3_^−^-N slightly increased to 0.98 ± 0.16 mg L^-1^ N and NO_2_^−^-N concentrations remained close to zero. Supplied SO_2_ gas was assessed as dissolved SO_4_^2-^, which accumulated equally between strains and the abiotic control; values reached 219 ± 33 and 207 ± 35 mg L^-1^ SO_4_^2-^ for HCA and LCA, respectively (Fig 3h).

### *De novo* transcriptome assembly and functional annotation

**Fig 4:**
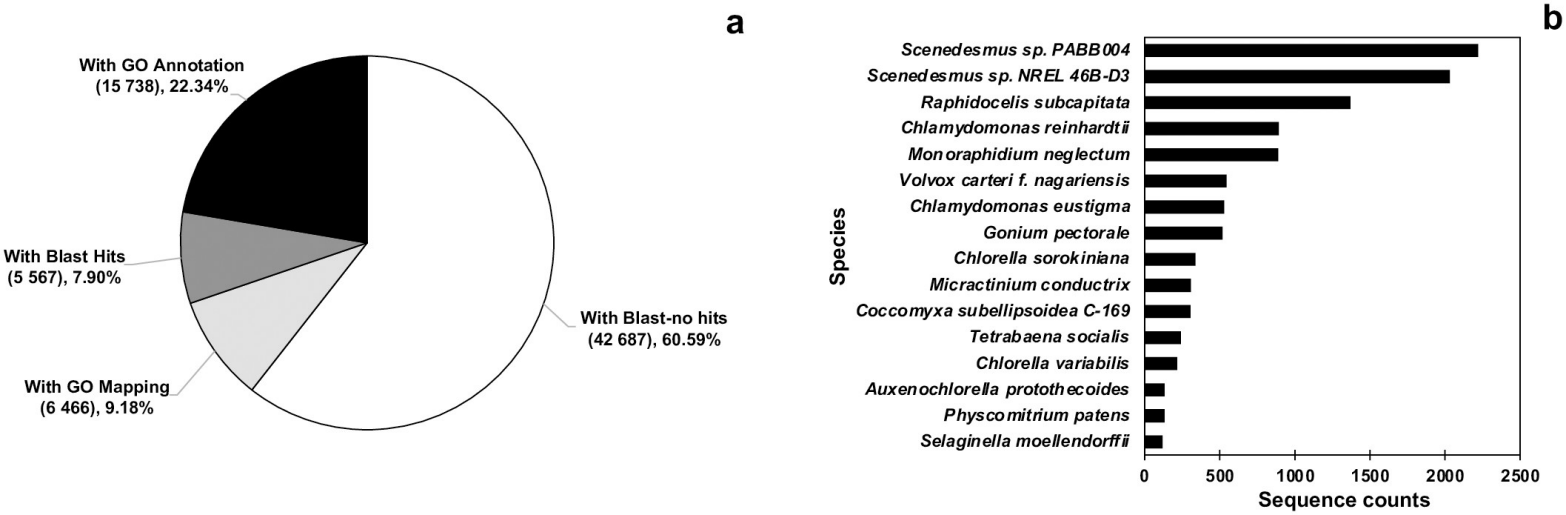
Functional annotation (a) and BLAST top-hit species (b) of the *de novo* transcriptome of *D*. *abundans* strains under model flue gas at day 4 (n = 3).

Transcriptome assembly resulted in 70 458 contigs with an average length of 881 bp and a 50^th^ percentile (N50) of 1 677 bp (Table 2, S1 Table). Completeness of the transcriptome was evaluated using BUSCO and the eukaryotic lineage dataset, which resulted in 277 out of 303 BUSCOs (91.4%), with 236 complete (77.9%) and 41 fragmented (13.5%) contigs. In addition, 91–92% alignment rates were obtained when the individual replicates of the strains were mapped to the assembled transcriptome. The proportion of redundant contigs determined in the CD-HIT-EST program was 0.37% and 0.01%, with a similarity of 95% and 100%, respectively. A total of 15 738 contigs (22.34%) were annotated with Gene Ontology (GO) terms, while 60.59% showed no hits (Fig 4a). BLAST top-hit species were *Scenedesmus* sp. strains PABB004 and NREL 46B-D3, and *Raphidocelis subcapitata* (Fig 4b).

**Table 2:** Statistic parameters and completeness of the *de novo* transcriptome assembly of *D*. *abundans* based on the Benchmarking Universal Single-Copy Orthologous (BUSCO) dataset.

**Assembly output**	**Value**
Total number of contigs	70 458
Min length (bp)	200
Max length (bp)	15 389
Average length (bp)	881
Standard deviation (bp)	1096
Median length (bp)	420
Total bases in contigs (bp)	62 071 384
Number of contigs < 500 bp	40 076
Number of contigs > or = 500 bp	30 382
Number of contigs > or = 1,000 bp	17 492
Number of contigs > or = 2,000 bp	7 894
Number of contigs > or = 5,000 bp	972
Number of contigs > or = 10,000 bp	27
N50 (bp)	1 677
Contigs in N50	60 352
GC content	55.60%
**BUSCO statistics**	**Value** ^a^
Complete BUSCOs	236 (77.9%)
Complete-single-copy BUSCOs	110 (36.3%)
Complete-duplicated BUSCOs	126 (41.6%)
Fragmented BUSCOs	41 (13.5%)
Missing BUSCOs	26 (8.6%)

^a^Considers 100 eukaryotes species and 303 BUSCOs [27].

In parenthesis, the percentage of BUSCOs in the assembled transcriptome.

### Differentially expressed genes between microalgae strains under model flue gas

**Fig 5:**
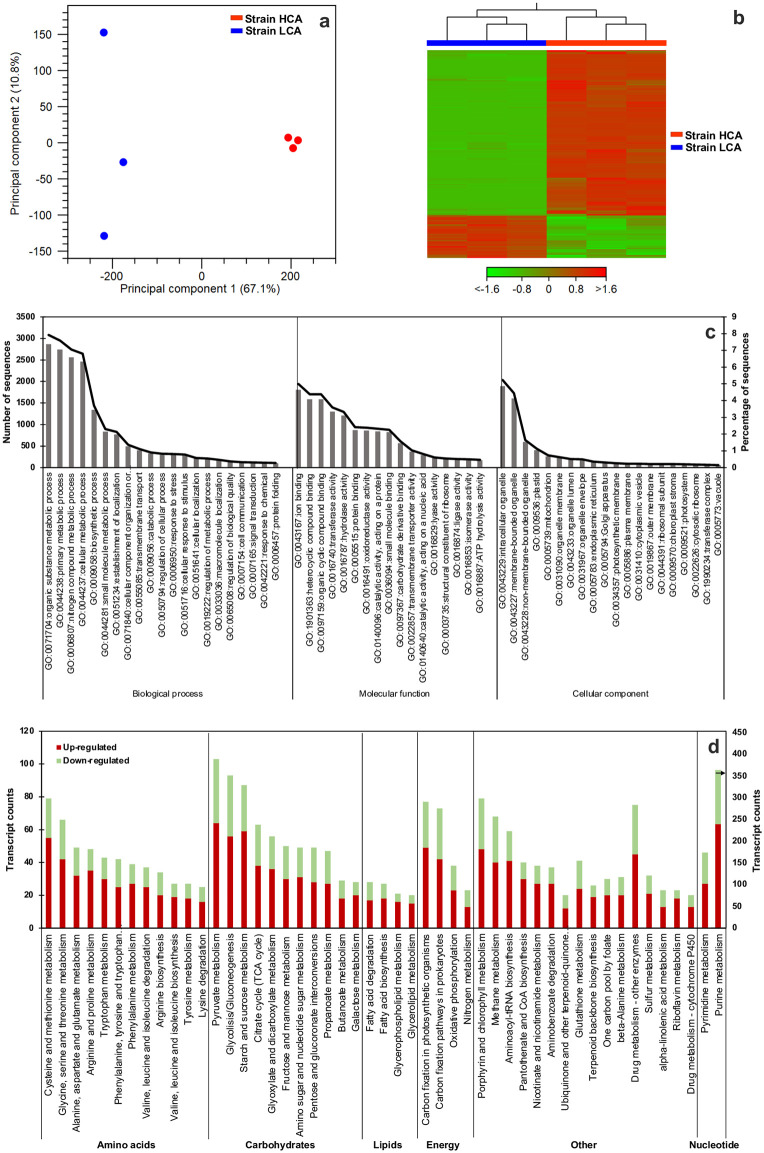
Principal component analysis (a), heatmap (b), gene ontology terms (c), and KEGG categories and subcategories of differently expressed genes in strain HCA compared to strain LCA (d) of the *de novo* transcriptome of *D*. *abundans* under model flue gas at day 4 (n = 3). Heatmap represents Z-score normalization of log CPM (counts per million) per transcript, FDR<0.01 and |log_2_ FC|>2. Color gradient represents the change in contig expression (red: up-regulated, green: down-regulated). Only KEGG categories with a contig count ≥ 20 are shown. Black arrow in purine metabolism indicates values on the secondary axis.

The principal component analysis (PCA) of gene expression separated the strains into two groups, which explained 67.1% of data variability (Fig 5a). Differential expression gene analysis revealed that strain HCA presented 16 435 up-regulated and 4 219 down-regulated genes compared to strain LCA (FDR < 0.01). The heatmap in Fig 5b reflects this finding by the normalized expression level of each sample and contig; most contigs were up-regulated in strain HCA. Also, it shows the hierarchical clustering of two groups that corresponded to each strain replicates.

Enrichment of DEGs with GO terms resulted in 7 995 (38.7%) contigs annotated, while 12 659 (24.0%) remained without annotation. From these, 16 809, 13 060, and 6 342 contigs were classified as genes participating in biological processes, molecular function, and cellular components, respectively (Fig 5c). Top biological process GO terms were organic substance metabolic process (7.9%), primary metabolic process (7.6%), and nitrogen compound metabolic process (7.1%); for molecular function, GO term corresponded to genes related to ion binding (5.0%), heterocyclic compound binding (4.4%), and organic cyclic compound binding (4.4%); and for cellular component GO term to intracellular organelle (5.2%) and membrane-bounded organelle (4.4%).

On the other hand, enzyme code and biochemical pathways were assigned to DEGs according to KEGG categories and subcategories (Fig 5d, S2 Table). The category with more DEGs was carbohydrates (654 DEGs), followed by amino acids (516 DEGs), nucleotides (410 DEGs), energy (211 DEGs), and lipids (96 DEGs) metabolism. For all categories, more contigs were up-regulated (1 628 of 2 529) in the HCA strain. As metabolisms of interest, carbon fixation pathways (in photosynthetic organisms and prokaryotes pathways) presented 91 up-regulated and 50 down-regulated contigs in the HCA strain. Fatty acid pathways showed 17 and 18 up-regulated genes in fatty acid degradation and biosynthesis, respectively, while 11 and 9 were down-regulated. For glycerophospholipid and glycerolipid metabolism, 16 and 15 contigs were up-regulated, and 5 contigs in both were down-regulated. Among amino acids, the pair cysteine and methionine metabolism presented the highest number of DEGs, with 55 up- and 24 down-regulated. Overall, the most changes per subcategory were in purine (239 up- and 125 down-regulated), pyruvate (64 up- and 39 down-regulated), glycolysis/gluconeogenesis (56 up- and 37 down-regulated), and starch and sucrose metabolism (59 up- and 28 down-regulated) (S2 File).

### Differentially expressed genes in central carbon metabolism

**Fig 6:**
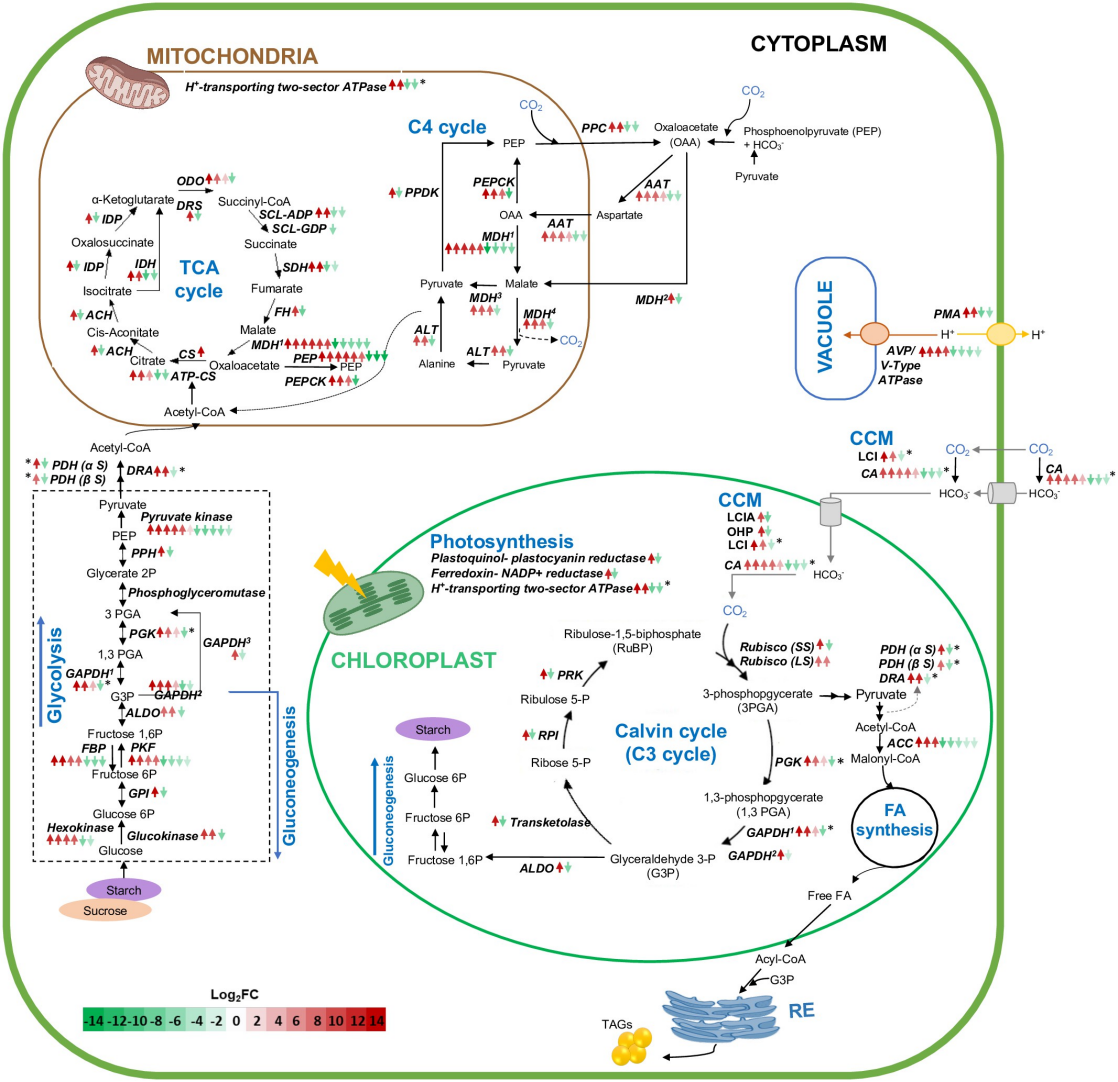
Differentially expressed genes in central carbon metabolism of the *de novo* transcriptome of *D*. *abundans* strain HCA compared to strain LCA under model flue gas at day 4. Arrows indicate number of contigs codifying for each enzyme or protein, and the color gradient correlates with expression level (red: up-regulated, green: down-regulated). Asterisks indicate contigs not annotated to a particular cellular compartment. Superscript numbers represent different forms of the enzyme in a specific pathway (details in S3 Table). CA: Carbonic anhydrase, LCI: Low CO_2_ inducible protein, LCIA: Low CO_2_ inducible protein A, OHP: Low CO_2_ and stress-induced one-helix protein, Rubisco: Ribulose-biphosphate carboxylase, PMA: H+-transporting ATPase, AVP: H^+^-translocating diphosphatase, PGK: Phosphoglycerate kinase, GAPDH: Glyceraldehyde 3-PO_4_ dehydrogenase, RPI: Ribose-5-phosphate isomerase, PRK: Phosphoribulokinase, PPC: Phosphoenolpyruvate carboxylase, AAT: Aspartate aminotransferase, MDH1: Malate dehydrogenase, PEPCK: Phosphoenolpyruvate carboxykinase, ALT: Alanine transaminase, PPDK: Pyruvate -phosphate dikinase, GPI: Glucose-6-PO_4_ isomerase, FBP: Fructose bisphosphatase, PFK: 6-phosphofructokinase, ALDO: Fructose-bisphosphate aldolase, PPH: Phosphopyruvate hydratase, PDH: Pyruvate dehydrogenase, DRA: Dihydrolipoyllysine- residue acetyltransferase, ACC: Acetyl-CoA carboxylase, CS: Citrate synthase, ATP-CS: ATP citrate synthase, ACH: Aconitate hydratase, IDH: Isocitrate dehydrogenase (NAD^+^), IDP: Isocitrate dehydrogenase, DRS: Dihydrolipoyllysine- residue succinyltransferase, SCL-GDP: Succinate- CoA ligase (GDP- forming), SCL-ADP: Succinate- CoA ligase (ADP- forming), SDH: Succinate dehydrogenase, and PEP: Phosphoenolpyruvate carboxykinase.

As mentioned above, transcriptome analysis revealed DEGs in several pathways of the central carbon metabolism, specifically in the C3 and C4 cycles, citrate cycle (TCA), and glycolysis and gluconeogenesis (Fig 6, S3 Table). Also, components of the carbon concentrating mechanism (CCM) and processes associated to CO_2_ tolerance were also included. For most enzymes and proteins, several unique contigs up- and down-regulated were found, but a higher number of contigs with higher magnitudes of change (log_2_FC) were up-regulated in the HCA strain. Annotation results showed presence of putative proton pumps associated to the plasma membrane (PMA) and vacuole (AVP/V-Type ATPase), which exhibited a higher magnitude of up-regulation in strain HCA (10.8 to 13.4 log_2_FC and -4.8 to -8.3 log_2_FC). Few components of the CCM presented DEGs, up-regulated to a greater extent in strain HCA and were annotated as putative carbonic anhydrases (CA), inorganic carbon transporters LCI and LCIA, and stress induced one-helix protein (OHP). Most of these contigs exhibited similarity to predicted proteins from other transcriptome studies in the database. Six out of eight contigs associated to CAs showed conserved domains to the alpha-type superfamily and, as the other DEGs, were mainly up-regulated in HCA (7.6 to 10.6 log_2_FC and -4.7 to -8.5 log_2_FC) (Fig 6, S3 Table). In the first step of CO_2_ fixation, ribulose-biphosphate carboxylase (Rubisco) subunits were identified with three up-regulated contigs (7.7 to 12.0 log_2_FC) and one down-regulated (-7.0 log_2_FC). Following, most enzymes in the Calvin-Beson-Bassham cycle (C3) were also differentially expressed with up to 15 log_2_FC, and those down-regulated ranged from -7.5 to -3.3 log_2_FC. These enzymes were phosphoglycerate kinase (PGK, EC:2.7.2.3), glyceraldehyde-3-phosphate dehydrogenases (GAPDH and NADP^+^, EC:1.2.1.12, EC:1.2.1.13, and EC:1.2.1.59), transketolase (EC:2.2.1.1), ribose-5-phosphate isomerase (RPI, EC:5.3.1.6), and phosphoribulokinase (PRK, EC:2.7.1.19).

Similarly, most enzymes in the Hatch-Slack cycle (C4) were differentially expressed with log_2_FC values of 3.6 to 13.7 for up-regulated and -13.9 to -4.8 for down-regulated contigs (Fig 6, S3 Table). These enzymes corresponded to phosphoenolpyruvate carboxylase (PPC, EC:4.1.1.31), aspartate aminotransferase (AAT, EC:2.6.1.1), malate dehydrogenases (MDH, EC:1.1.1.37, EC:1.1.1.82, EC:1.1.1.40 and EC:1.1.1.39), phosphoenolpyruvate carboxykinase (ATP) (PEPCK, EC:4.1.1.49), alanine transaminase (ALT, EC:2.6.1.2), and pyruvate-phosphate dikinase (PPDK, EC:2.7.9.1).

Also, three enzymes participating in photosynthesis were differentially expressed: plastoquinol- plastocyanin reductase (one up- and one down-regulated), ferredoxin- NADP^+^ reductase (one up- and one down-regulated), and H^+^ -transporting two-sector ATPase (two up- and two down-regulated). GO terms of contigs codifying for cellular components related to photosynthesis showed a more pronounced up-regulation rather than down-regulation (S1 Fig). DEGs of chloroplast components (stroma, envelope, and thylakoid membrane) comprised around 30 to 40 up- and down-regulated contigs, where up-regulation ranged from 8 to 14 log_2_FC and down-regulation from -8 to -2 log_2_FC (S1a Fig). For photosystems, 48 DEGs were identified as photosystem I, of which 28 were up-regulated and 20 down-regulated, and for photosystem II, 57 DEGs, 35 up-regulated and 22 down-regulated (S1b Fig) with log_2_FC values of 11 to 14 and 8 to 14 for photosystem I and II, respectively, and down-regulation of -8 to -2 and -7 to -2.

In addition, most enzymes involved in glycolysis and gluconeogenesis were also differentially expressed, whose log_2_FC values range from 2.3 to 15.4 and from -8.8 to -2.0 for up- and down-regulated contigs, respectively. In the TCA cycle, up-regulated genes reached 15.1 log_2_FC, and most down-regulated were between -10.8 and -4.3 log_2_FC. Likewise, three enzymes responsible for the conversion of pyruvate to malonyl-CoA were identified with three up- and two down-regulated contigs for pyruvate dehydrogenase (PDH, EC:1.2.4.1), two up- and one down-regulated for dihydrolipoyllysine-residue acetyltransferase (DRA, EC:2.3.1.12), and three up- and five down-regulated for acetyl-CoA carboxylase (ACC, EC:6.4.1.2).

### Differentially expressed genes in one carbon, starch, and triacylglycerol metabolism

**Fig 7:**
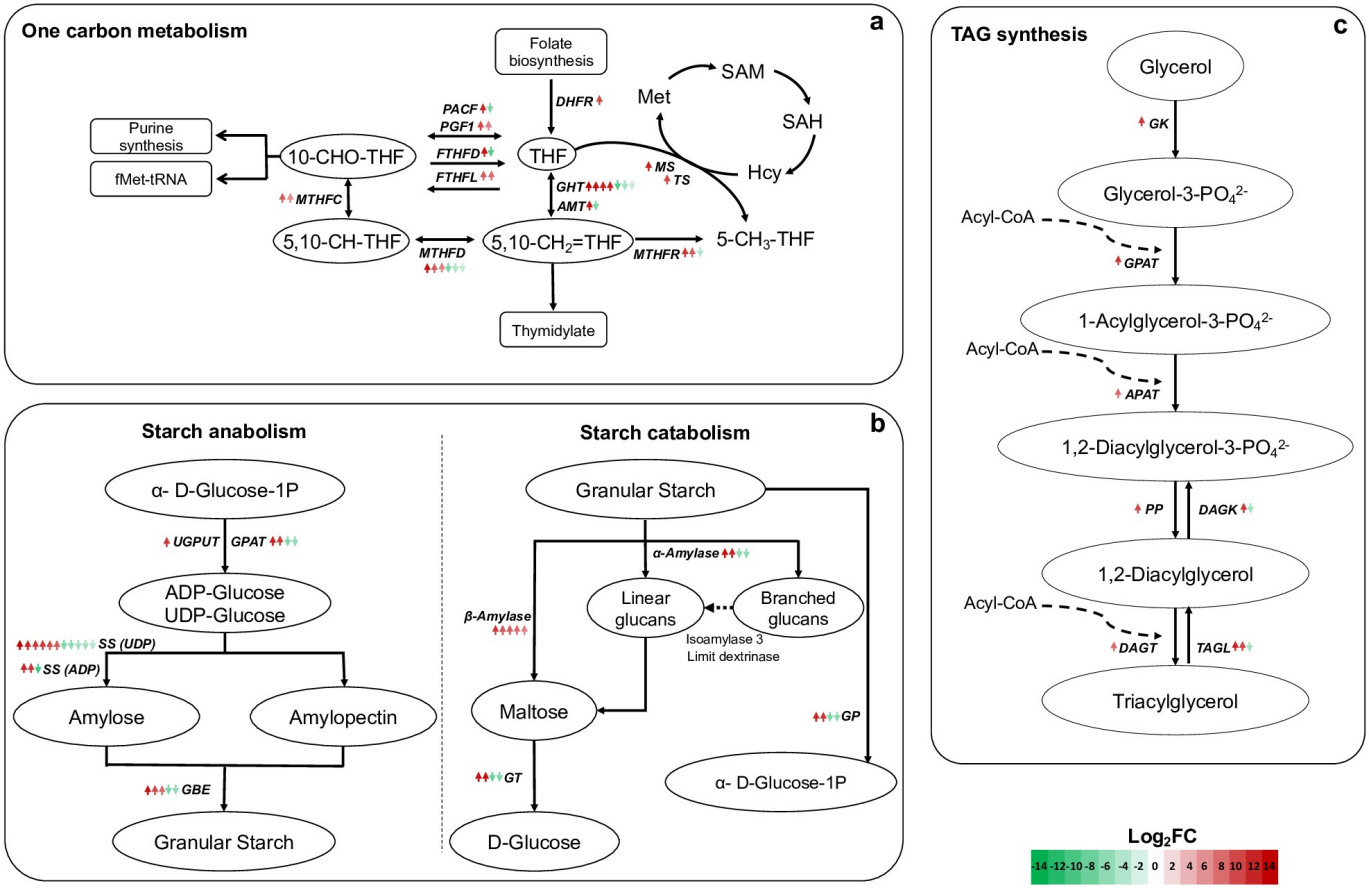
Differentially expressed genes in one-carbon metabolism (a), starch anabolism and catabolism (b), and triacylglycerol synthesis (c) of the *de novo* transcriptome of *D*. *abundans* strain HCA compared to strain LCA under model flue gas at day 4. Arrows indicate number of contigs encoding for each enzyme, and the color gradient correlates with expression level (red: up-regulated, green: down-regulated). PGF1: Phosphoribosylglycinamide formyltransferase 1, PACF: Phosphoribosylaminoimidazolecarboxamide formyltransferase, FTHFL: Formate- tetrahydrofolate ligase, FTHFD: Formyltetrahydrofolate deformylase, MTHFC: Methenyltetrahydrofolate cyclohydrolase, MTHFD: Methylenetetrahydrofolate dehydrogenase (NADP+), MTHFR: Methylenetetrahydrofolate reductase (NADPH), GHT: Glycine hydroxymethyltransferase, DHFR: Dihydrofolate reductase, AMT: Aminomethyltransferase, MS: Methionine synthase, TS: Thymidylate synthase, GPAT: Glucose-1-phosphate adenylyltransferase, SS (ADP): Starch synthase (glycosyl-transferring), SS (UDP): Glycogen (starch) synthase, GBE: 1,4-α-glucan branching enzyme, GT: 4-α-glucanotransferase, GP: Glycogen phosphorylase, GK: Glycerol kinase, GPAT: Glycerol-3-phosphate acyltransferase, APAT: 1-acylglycerol-3-phosphate O-acyltransferase, DAGK: Diacylglycerol kinase (ATP), PP: Phosphatidate phosphate, DAGT: Diacylglycerol O-acyltransferase, and TAGL: Triacylglycerol lipase.

DEGs of enzymes in one carbon by folate metabolism were studied in detail as an essential cofactor related to purine biosynthesis, the latter with the highest differently expressed genes (Fig 7a, S2 File a). Folate metabolism exhibited contigs up- (5.8 to 14.1 log_2_FC) and down-regulated (-9.3 to -2.6 log_2_FC), such as formyltetrahydrofolate deformylase (FTHFD), methylenetetrahydrofolate dehydrogenase (NADP^+^) (MTHFD), methylenetetrahydrofolate reductase (NADPH) (MTHFR), glycine hydroxymethyltransferase (GHT), and aminomethyltransferase (AMT) (Fig 7a, S4 Table). In addition, some enzymes presented only up-regulated contigs: phosphoribosylglycinamide formyltransferase 1 (PGF1, 7.3 to 10.4 log_2_FC), formate-tetrahydrofolate ligase (FTHFL, 7.9 to 8.6 log_2_FC), methyltetrahydrofolate cyclohydrolase (MTHFC, 5.8 to 8.6 log_2_FC), dihydrofolate reductase (DHFR, 10.0 log_2_FC), methionine synthase (MS, 11.6 log_2_FC), and thymidylate synthase (TS, 10.0 log_2_FC).

In addition, routes of interest in secondary metabolism were also analyzed. DEGs of key enzymes involved in starch anabolism showed contigs up- (7.9 to 13.5 log_2_FC) and down-regulated (-9.6 to -3.7 log_2_FC), such as glucose-1-phosphate adenylyltransferase (GPAT), starch synthases (SS-UDP and SS-ADP), and 1,4-α-glucan branching enzyme (GBE) (Fig 7b; S4 Table). UTP-glucose-1-phosphate uridylyltransferase (UGPUT) presented only one contig up-regulated (11.4 log_2_FC). On the other hand, in starch catabolism, β-amylase presented five up-regulated contigs (7.5 to 11.31 log_2_FC). At the same time, α-amylase, 4-α-glucanotransferase (GT), and glycogen phosphorylase (GP) showed two up- (9.6 to 12.2 log_2_FC) and two down-regulated contigs (-6.9 to -6.2 log_2_FC).

Enzymes involved in triacylglycerol (TG) biosynthesis were up-regulated (7.6 to 10.9 log_2_FC) and contemplated glycerol kinase (GK), glycerol-3-PO_4_ acyltransferase (GPAT), 1-acylglycerol-3-PO_4_ O-acyltransferase (APAT), phosphatidate phosphate (PP), and diacylglycerol acyltransferase (DAGT) (Fig 7c, S4 Table). Also, contigs for enzymes related to TG degradation were up- (10.7 to 11.6 log_2_FC) and down-regulated (-5.2 to -4.6 log_2_FC): triacylglycerol lipase (TAGL) and diacylglycerol kinase (DAGK), respectively.

### Differentially expressed genes in nitrogen transport and metabolism

**Fig 8:**
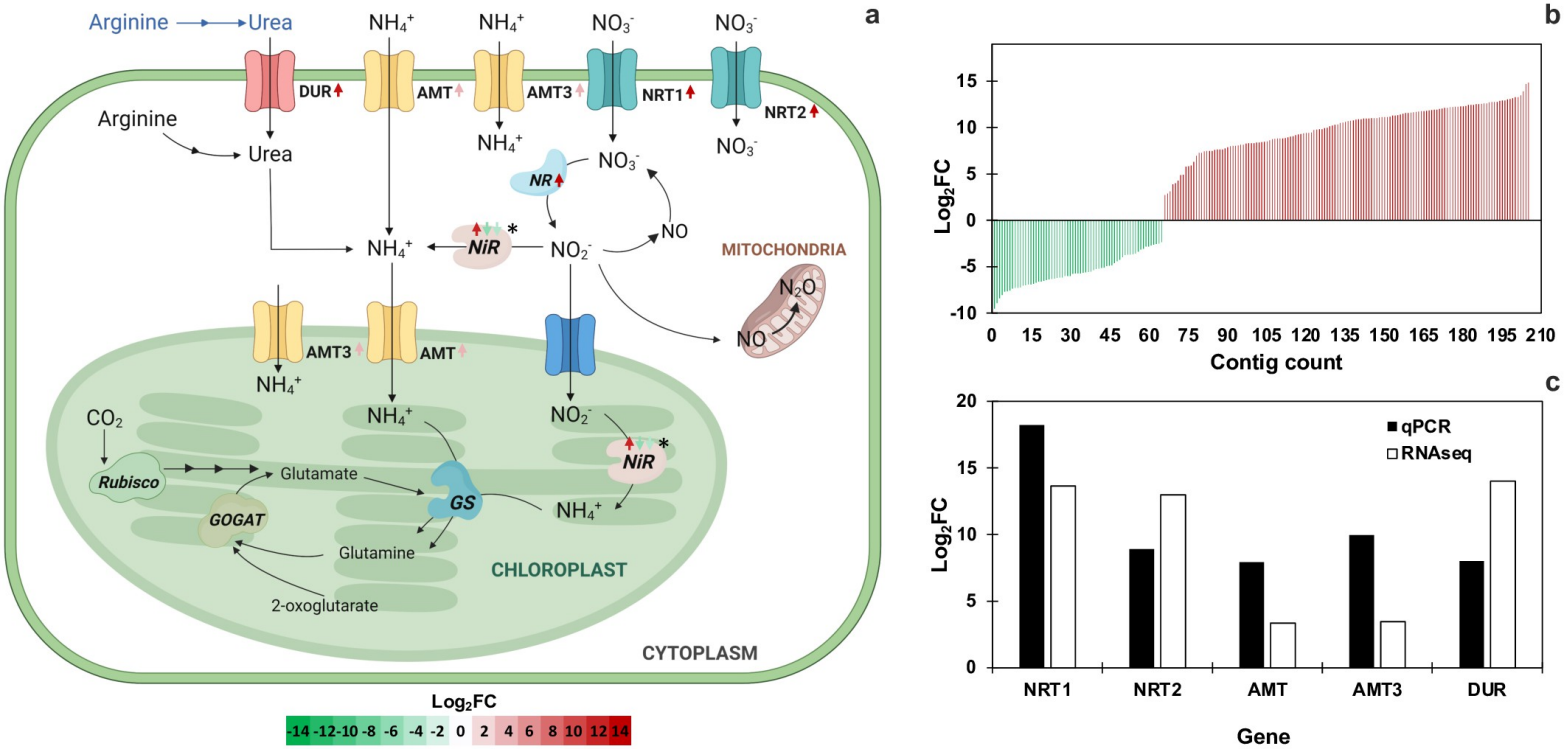
Scheme proposed for *D*. *abundans* nitrogen assimilation (a), expression level (log_2_FC) of contigs annotated as nitrogen transporter GO term (b), and validation of gene expression using RT-qPCR (c). Arrows indicate number of contigs codifying for each enzyme, and the color gradient correlates with expression level (red: up-regulated, green: down-regulated). GS: glutamine synthase, GOGAT: glutamate synthase, NR: nitrate reductase, NiR: nitrite reductase, DUR: urea transporter, AMT: ammonium transporter, NRT: nitrate transporter, CA: carbonic anhydrase. Asterisks indicate contigs not annotated to a particular cellular compartment. Adapted from Kumar and Bera (2020), Bellido-Pedraza *et al*. (2020), Scherholz and Curtis (2013), Sanz-Luque *et al*. (2015), and Dong *et al*. (2014) [39–43].

Since strains showed differential capability to scavenge N from MFG in the incomplete culture medium, contigs annotated in transport and N metabolism were further analyzed. DEGs analysis showed a significant amount of contigs identified as nitrogen transporters, specifically of nitrate (NTR), ammonium (AMT), and urea (DUR) (Fig 8a). Also, a contig encoding for nitrate reductase (NR) exhibited up-regulation of 14.3 log_2_FC in strain HCA compared to LCA, and the nitrite reductase (NiR) exhibited one contig up- (11.9 log_2_FC) and two down-regulated (-5.0 to -3.3 log_2_FC) (Fig 8a, S5 Table). Nitrogen transporter GO term comprised a total of 140 contigs up-regulated (2.7 to 15 log_2_FC) and 65 down-regulated (-2.4 to -9.5 log_2_FC) in strain HCA (Fig 8b).

As a route of interest in NOx utilization, the expression of these genes was validated using RT-qPCR (Fig 8c). All nitrogen transporters were up-regulated when comparing strain HCA with LCA in the RNASeq analysis. RT-qPCR confirmed this pattern where NRT1 and NRT2 were up-regulated by 18.0 and 8.9 log_2_FC, respectively, and AMT and AMT3 by 7.9 and 10.0 log_2_FC. Finally, DUR also showed upregulation by 8.0 log_2_FC.

### Lipidome analysis

**Fig 9:**
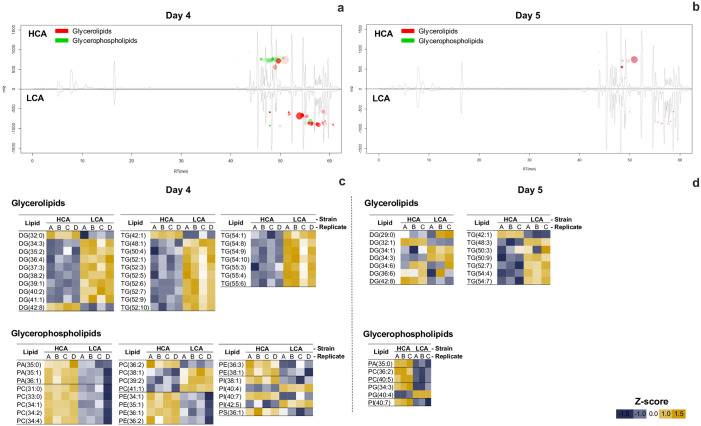
Differential lipidome analysis of *D*. *abundans* strain HCA compared to strain LCA under model flue gas at day 4 and 5 (t-test, FDR<0.05, n = 3). Representative chromatograms with cloud plots indicate molecules with significant differences at day 4 (a) and 5 (b). Dots in negative m/z represent features that decreasedDot size and color intensity represent magnitude of log_2_FCand significance of difference. Heatmap of the log-transformed intensity of glycerolipids and glycerophospholipids at day 4 (c) and 5 (d). Values represent Z-scores per row. Color gradient represents the change in feature intensity (yellow: high, blue: low). DG: diacylglycerols, TG: triacylglycerols, PA: glycerophosphates, PC: glycerophosphocholines, PE: glycerophosphoethanolamines, PI: glycerophosphoinositols, and PS: glycerophosphoserines. In parenthesis, side-chain carbon number and unsaturation degree.

Strains’ lipidome were also analyzed at day 4 as the transcriptome, and at early stationary phase on day 5 (Fig 9). Cloud plots in representative chromatograms at day 4 showed a higher density of putative glycerophospholipids (GP) in strain HCA compared to LCA andglycerolipid (GL) intensities were mainly higher in strain LCA (Fig 9a). On the other hand, cloud plots at day 5 showed a lower number of lipids with significant differences between strains (Fig 9b).

GL features at day 4 corresponded to diacylglycerols (DG) and triacylglycerol (TG) that mostly decreased in strain HCA compared to LCA (Fig 9c). Features matching GP m/z were identified as three glycerophosphates (PA), nine glycerophosphocholines (PC), six glycerophosphoetanolamines (PE), four glycerophosphoinositol (PI), and one glycerophosphoserine (PS) (Fig 9c). Of these, all PA, all PE, all PS, six PC, and two PI showed higher intensities in strain HCA.

As previously mentioned, a lower number of significant features was observed at day 5 but lipid patterns remained (Fig 9d). Most putative DG and TG intensities were lower in strain HCA and, in contrast, GP showed higher intensities except for PG 40:4. The latter feature only assessed at day 5 together with PG 34:3. Overall, putative DG were characterized by 29 to 42 C side-chains and TG from 42 to 55 C. Most DG and TG were unsaturated with up to 10 double bonds, except for one saturated DG (29:0 and 32:0). On the other hand, putative GP were predicted to have C side-chains of 31 to 42 and 0 to 7 unsaturations, except PA (35:0) and PC (31:0 and 33:0).

### Cell structure and biomass composition

**Fig 10:**
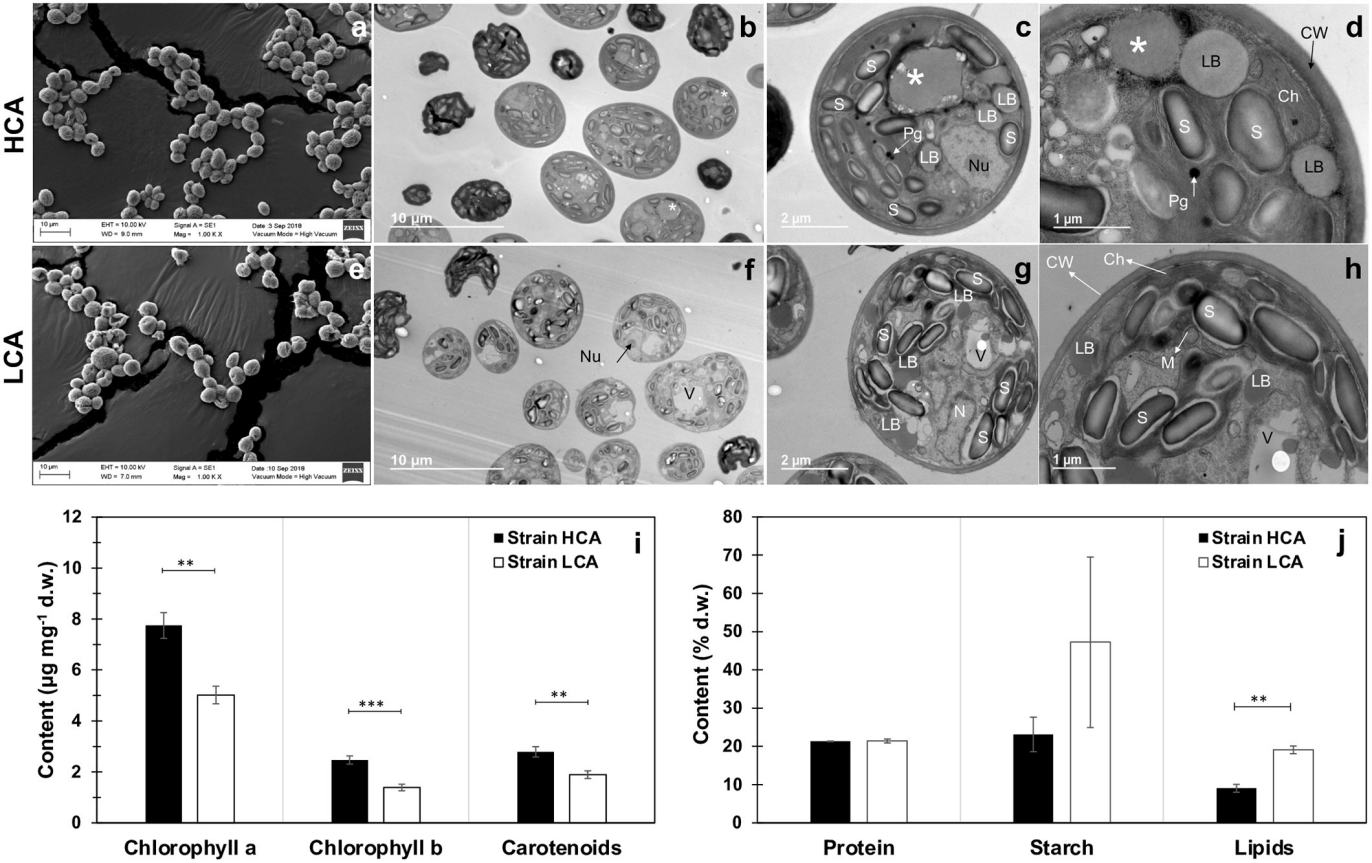
Cell structure and biomass composition of *D*. *abundans* strain HCA compared to strain LCA under model flue gas at day 5. Scanning and transmission electron micrographs of strain HCA (a-d) and LCA (e-h). Biomass pigments (i) and protein, starch, and lipid content (j). Ch, chloroplast; M: mitochondria; N: nucleus; Nu: nucleolus; V: vacuole; S: starch grain; LB: lipid body; P: pyrenoid; Pg: plastoglobuli; Pp: polyphosphate granule; CW: cell wall; white asterisk: large spherical organelle.

Most cells of both strains under MFG presented a unicellular circular morphology (Fig 10a–10h), contrary to typical linearly ordered *Desmodesmus* arrangements of two to eight ellipsoidal cells with spines in the outer cells. According to SEM and TEM information, cell size under MFG did not differ between strains with averages of 6.4 ± 1.3 and 6.1 ± 1.1 μm of diameter, and 29.1 ± 10.1 and 27.0 ± 10.2 μm^2^ of cell area for strain HCA and LCA, respectively (t-test, α = 0.05, n = 35). However, a thicker cell wall (1.6 times) was observed in strain HCA (203.8 ± 43.9 nm) compared to LCA (128.2 ± 18.9 nm) (t-test, α = 0.05, n = 48) (Fig 10d–10h). TEM images showed in both strains a great quantity of starch grains, several lipid bodies and plastoglobuli, and a disorganized chloroplast containing a high amount of storage structures (Fig 10b–10d and 10f–10h). A large unidentified spherical organelle was observed in strain HCA (Fig 10d), while strain LCA showed large vacuoles (Fig 10h).

In addition, differences among strains’ biomass composition were observed under MFG. First, strain HCA presented a significant higher concentration of chlorophylls (*Chl*) and carotenoids, around 1.4 times (t-test, α = 0.05, n = 6) (Fig 10i). As expected, *Chl a* content was the highest (2.8 to 3.1 times) among pigments. For example, strain HCA possessed 7.7 ± 0.5 μg mg^-1^ *Chl a*, 2.5 ± 0.2 μg mg^-1^ *Chl b*, and 2.8 ± 0.2 μg mg^-1^ carotenoids. However, protein content was similar between strains, around 21% d.w. (Fig 10j). On the other hand, starch content was 23.1 ± 4.5 and 47.2 ± 22.3% d.w. for strain HCA and LCA, respectively (Fig 10j). Even though strain LCA exhibited a highly variable starch content, on average this value was two-fold strain’s HCA. Comparably, lipid content was higher in strain LCA, 19 ± 1 compared to 9 ± 1% d.w. (t- test, α = 0.05, n = 6) (Fig 10j).

## Discussion

### Phylogenetic analysis revealed differences between *D*. *abundans* strains after 10 years of acclimation to low and high CO_2_

The identity of both strains was confirmed as *D*. *abundans* (Fig 1c and 1d). However, strain HCA was located at a different position and distance from strain LCA in both phylogenetic analyses, which suggests that the HCA strain has diverged from LCA over the past 10 years due to the high CO_2_ acclimation strategy, which represents the only difference between microalgae growth conditions.

Transcriptome analysis also confirmed that both strains belong to the Scenedesmaceae family. Still, at the genus or species level, there were no hits with *D*. *abundans* due to the lack of available information. Specifically, Blast top-hit species to *D*. *abundans* transcriptome were *Scenedesmus* spp. (Fig 4b). The highest hits (2 221 sequence counts) were with the *Scenedesmus* sp. strain PABB004, followed by *Scenedesmus* sp. NREL 46B-D3 (2 034 sequence counts). Also, species from the Selenastraceae family presented high Blast hits, *Raphidocelis subcapita* (1 371 hits), and *Monoraphidium neglectum* (890 hits). Scenedesmaceae and Selenastraceae are families from the Sphaeropleales order. Other microalgae species contributing to the transcriptome annotation were *Chlamydomonas reinhardtii*, *Volvox carteri*, *Chlamydomonas eustigma*, and *Gonium pectoral* from the Chlamydomonadales order, also known as Volvocales. Similar results have been obtained for the *de novo* transcriptome assembly of other microalgae species from the Scenedesmaceae family [17]. However, these results depend on available information, which is still limited in microalgae studies. As observed from functional annotation results, only 39.41% of contigs presented Blast hits (Fig 4a). Hanschen and Starkenburg [19] mentioned the necessity of generating more species genomes and gene annotations with higher quality and accessibility to gene models.

### Microalgae acclimated to high CO_2_ present higher tolerance to model flue gas

After 10 years, the strains’ tolerance and capability to grow and use MFG as a nutrient source were evaluated under continuous gas supply. Also, it was confirmed that the strains possess different upper tolerable CO_2_ concentrations, where strain HCA was able to grow at 75% CO_2_ contrary to strain LCA (Fig 1b). Under MFG with 25% CO_2_, both strains presented a lag phase of 24 h during the adjustment period. However, strain HCA started growing at a higher rate (1.9 times from 24 to 48 h) and entered the stationary phase a day earlier than strain LCA (Fig 3a and 3b, Table 1). By the end of the experimental period, and probably due to photobioreactor limitations, both cultures showed similar cell concentrations (2.5 ± 0.7 and 2.3 ± 0.8 x 10^7^ cells mL^-1^ for HCA and LCA, respectively) and biomass productivities (0.33 0.06 and 0.34 0.13 g L^-1^ d^-1^). Nevertheless, strain HCA reached maximums a day before strain LCA, and growth seemed limited after day 2, probably due to low N supply by MFG NO_x_ content. Further analysis under a continuous growth scheme should exploit HCA’s initial growth rate and determine if additional N is necessary to achieve optimal performance.

Both strains demonstrated the capacity to use MFG as the sole source of C, N, and S. Cement flue gas contains considerable amounts of CO_2_, NO_x_, and SO_x,_ which can serve as feedstocks for microalgae cultivation [2]. Specifically, NO and SO_2_, as the main components of NO_x_ and SO_x_, can be oxidized in aerobic conditions to nitrite or nitrate and sulfate, which can be used by microalgae [6, 44]. These components, together with CO_2,_ can be used directly from industry and converted to valuable byproducts. Different studies evidence this potential, where microalgae tolerate, grow, and generate compounds of industrial interest [4, 45–48].

In the present study, microalgae tolerance and utilization of MFG as the sole N source were evident when analyzing dissolved concentrations of nitrate and nitrite in the culture medium, which correlated with the growth patterns observed for each strain (Fig 3a, 3b, 3f and 3g). In strain HCA, dNO_x_ values remained below 1 mg L^-1^ N during the experimental period. However, for strain LCA that initially grew slower, dNO_x_ accumulated in the culture medium until day 3 and then decreased in accordance with late exponential growth. N is an essential nutrient used for *de novo* protein synthesis, which is crucial during exponential growth [49]. In addition, dNO_x_ concentrations in abiotic versus biotic conditions exemplified the potential and assimilation of N from MFG by the microalgae, which reached N utilization efficiencies of 100% based on biomass productivity. Still, the water solubility of NO is low, and it is considered a limiting step of the system [50]. On the contrary, dSO_x_ concentrations were not different from the abiotic control, probably because of the high solubility of SO_2_ in water and the continuous supply by the MFG (Fig 3h). Based on biomass production, S utilization efficiencies of the strains were 35 to 38%.

In this study, adding CDK to the photobioreactor effectively maintained the pH close to 6.5 (Fig 3c). This is a critical consideration because at low pH levels (pH <5), SO_x_ is present in the form of bisulfate, which can be damaging to microalgae. When microalgae assimilate bisulfate and convert it to SO_4_^2-^, oxidative species are formed that can damage membranes and pigments and inhibit growth [20, 51]. Therefore, controlling pH is essential in increasing microalgae tolerance to flue gas, and using phosphate buffer and CaCO_3_ are some of the implemented methods [38, 46, 48]. However, these strategies compromise the simplicity and economy of the system [6]. Because CKD is a byproduct of the cement production process and represents a buffering agent of no cost generated during the calcination process [52], it has been proposed to control culture pH [6, 20, 53].

Finally, dCO_2_ increased consistently to a maximum average of 30 to 32% by day 5 (Fig 3d). Under the bioreactor pH, which ranged from 6 to 7, most dissolved inorganic carbon was in the form of HCO_3_^-^_,_ and only a small part was CO_2_. Both forms can be captured by microalgae [54]. According to biomass production, CO_2_ fixation rates were similar between strains at the end of the experimental period. Oxygen production correlated with biomass production and reached comparable values between strains (107% dO_2_ saturation) (Fig 3e).

### Highly up-regulated genes in central carbon metabolism under model flue gas show adaptative changes of the high CO_2_ acclimated strain

The adaptability of the HCA strain to MFG was reflected in the number of up-regulated contigs of diverse pathways (16 435 vs. 4 219 down-regulated) compared to strain LCA (Figs 5–8). Therefore, strain HCA might possess a higher capacity to respond to stressful conditions because of the acclimation strategy to high CO_2_ atmospheres for 10 years. As seen in Fig 6, strain HCA and LCA possessed several contigs for most genes in all pathways (S1 and S3 Tables), probably due to acquired genome differences during the acclimation strategy or differential preference of gene isoforms between strains.

Most DEGs up-regulated were related to microalgae growth, such as nucleotide metabolism (particularly purine metabolism), amino acid metabolism, central carbon metabolism, and carbon fixation, suggesting that strain HCA was actively growing under MFG (Figs 5 and 6, S2 File). In addition, tolerance to high CO_2_ of HCA might be associated with the capacity to control intracellular acidification, as plasma membrane and vacuole proton pumps were up-regulated to a higher extent in this strain (Fig 6). In the presence of an acidic gas, a higher concentration of protons is expected because of CO_2_ hydration in the extracellular and internal cell environment [55]. Choi *et al*. [55] demonstrated that plasma membrane pumps are a genetic engineering-based strategy to increase high CO_2_ tolerance in microalgae. Other expected cell response is the down regulation of the CCM not only because it is known that most of its components in microalgae are down-regulated by high CO_2_ [56] but also to inactivateCO_2_ hydration catalyzed by CA and control internal pH [55]. On the contrary, our study showed some CCM components up-regulated to a greater extend in the HCA strain under high CO_2_. Of these, putative CAs possessed conserved regions to the alpha-type family from which some are associated or possess constitutive expression at high CO_2_ [56]. Therefore, they might be active to assist other metabolic pathways. For example, N assimilation in dinoflagellates using cyanate, a breakdown product of internally produced urea, is degraded by cyanase in a HCO_3_^-^ dependent pathway to generate ammonia [57]. Hence, a CA is involved to generate HCO_3_^-^ from CO_2_ hydration. Since our condition was limited in N this might be a strategy to assist rapid growth under high CO_2_. Cyanase (EC:4.2.1.104) was found in our transcriptome up-regulated by 3.4 log_2_ FC in HCA (S1 Table). This enzyme has not been formerly reported in green algae but there are 19 entries in the NCBI database. Similarly, inorganic C-transporter LCI, putatively identified and located in the chloroplast, and LCIA are reported to be associated with a low CO_2_ condition [56]. LCIA can also transport nitrite and present a differential response to changes in the nitrogen-carbon cell condition and be regulated by the nitrate regulatory gene Nit2. In accordance, we did not observe a corresponding up-regulation of the transcription activator CIA5 known to be essential for expression of Ci transporters in *Chlamydomonas* [56].

Other up-regulated pathways of interest, such as the one-carbon metabolism by folate (Fig 7a), generate essential cofactors (e.g., 10-CHO-THF) in nucleotide synthesis, growth stimulation, and generation of reducing power [5, 10]. In agreement, Shin *et al*. [10] found in *Dunaliella tertiolecta* under N depletion a correlation between the down-regulation of genes from one-carbon by folate metabolism and growth inhibition. In general, strain HCA and LCA possessed several contigs for most genes in all pathways, probably due to acquired genome differences during the acclimation strategy or differential preference of gene isoforms between strains.

Pathways of central carbon metabolism were highly up-regulated in strain HCA compared to LCA (Fig 6). In the C3 cycle, most enzymes presented several contigs with differential expression. Still, a higher number of up-regulated genes were observed, which also presented a higher magnitude of change than those down-regulated. Associated with the C4 cycle, PPC is a light-dependent enzyme that converts phosphoenolpyruvate to oxaloacetate by fixation of CO_2_ and is typically overexpressed under high light intensity. MDH catalyzes the conversion of oxaloacetate to malate, which is a reversible reaction. Oxalacetate is a substrate used in gluconeogenesis, urea, amino acid synthesis, and TCA cycles. Pyruvate from malate conversion can then be transformed into acetyl-CoA [10]. The acetyl-CoA obtained from glycolysis or the C4 cycle can enter the TCA cycle to produce NADH and generate ATP by oxidative phosphorylation; also, the intermediates (α-ketoglutarate, succinyl-CoA, fumarate, and oxaloacetate) can be used in amino acid metabolism, while malate can be converted to pyruvate and then used in fatty acid synthesis [15].

Additionally, enzymes of glycolysis and gluconeogenesis and TCA cycle presented also contigs up-regulated in strain HCA and, to a lesser degree, contigs also down-regulated. Sun *et al*. [15] also observed up-regulation of genes related to the TCA cycle and carbohydrate metabolism when *Chlorella sorokiniana* was cultured under high CO_2_. Therefore, general up-regulation of these pathways in strain HCA compared to LCA was probably induced by the ability of HCA to provide and utilize internally a higher carbon flux from MFG.

### Strain-dependent gene regulation in starch and triacylglycerol metabolism triggered by limited N and high CO_2_ under model flue gas

Nitrogen and sulfur are essential nutrients for protein biosynthesis, lipids, chlorophyll, and photosystem proteins, among others. Usually, under N limitation, microalgae degrade chlorophyll, carotenoids, and thylakoid membrane macromolecules for nutrient acquisition; at the same time, growth is retarded, and storage compounds start to accumulate [16, 58, 59]. Under MFG and incomplete culture medium, N appears to be a limiting factor for growth, especially for strain HCA, since the culture reached stationary phase earlier (Fig 3b) and low dissolved N concentrations were observed throughout the experimental period (Fig 3f and 3g). As a response, genes of enzymes participating in synthesizing and degradation of storage metabolites such as starch and TG were differentially expressed between strains (Fig 7b and 7c). For these pathways, particularly for starch metabolism, different contigs were expressed between strains; however, a higher number of up-regulated contigs with a high magnitude of change were found in strain HCA.

In starch anabolism, the different enzymes catalyzing the conversion of α-D-Glucose-1P into starch were found up- and some down-regulated in strain HCA compared to LCA, except UTP-glucose-1-phosphate uridylyltransferase (UGPUT) that only showed one contig up-regulated in strain HCA. On the other hand, in starch catabolism, α-amylase, glycogen phosphorylase (GP), and 4-α-glucanotransferase (GT) were highly up-regulated in strain HCA and down-regulated to a lesser degree, while β-amylase presented five up-regulated contigs. Tan *et al*. [58] observed that *Dunaliella tertiolecta* under N depletion accumulated starch and up-regulated genes in starch biosynthesis and the TCA cycle, revealing an active interchange of carbon skeletons for anabolic and catabolic processes. Similarly, in this study, genes from starch and TCA cycle were up-regulated in strain HCA and, as mentioned above, probably because of a higher C flux internally.

As observed in Fig 6, three enzymes in fatty acid metabolism responsible for the conversion of pyruvate to malonyl-CoA were differentially expressed between strains. Pyruvate dehydrogenase (PDH) and dihydrolipoyllysine-residue acetyltransferase (DRA) exhibited a higher fold change of up-regulated contigs in strain HCA, but acetyl-CoA carboxylase (ACC) showed the opposite pattern (Fig 6). Acetyl-CoA from catalysis of pyruvate by PDH links glycolysis with lipid biosynthesis pathways, then ACC catalyzes conversion to malonyl-CoA, being these the first steps for *de novo* fatty acid biosynthesis [60]. Because of the low concentration of N in the medium, acetyl-CoA, commonly used to support algal growth by entering the TCA cycle under N-replete conditions, could be relocated to the chloroplast to generate storage compounds [13]. Also, under high CO_2,_ it has been observed that the flux of acetyl-CoA increases resulting in lipid accumulation [15, 61]. Higher up-regulated ACC enzyme contigs in strain LCA suggest that this strain redirects more carbon to lipid synthesis than strain HCA, probably because flue gas represents a higher stress condition for the air-acclimated strain.

However, enzymes from the final steps of TG biosynthesis were up-regulated in strain HCA, and only one contig characterized each enzyme (Fig 7c). Several studies have reported TG accumulation when grown under nutrient limitation [13, 14, 61, 62]. Despite higher expression of contigs in TG synthesis for strain HCA, the corresponding lipidome on day 4 showed that strain LCA possessed a higher concentration of TG (Fig 9a and 9c), which could be explained because enzymes related to TG degradation were also up-regulated in HCA. Triacylglycerol lipase (TAGL) and diacylglycerol kinase (DAGK) showed two contigs up-regulated with higher expression levels than those participating in synthesis (10.0 to 11.6 vs. 7.6 to 10.9 log_2_FC). Therefore, this suggests that degradation occurred faster than synthesis in strain HCA. Lipases can be found in membranes and lipid droplets; their function is to cleave FA from glycerol in TG molecules, then the degradation of FA to acetyl-CoA occurs, and acetyl-CoA enters the glyoxylate cycle and gluconeogenesis [63]. Lipid accumulation in *Scenedesmus acutus* under N starvation has been suggested to occur by inhibiting TG turnover and down-regulation of TG and DG lipases [17]. According to Kong *et al*. [63], there is still much unknown about microalgae lipid catabolism and enzyme identities.

### Differentially expressed genes of cellular components of the chloroplast and nitrogen transporters as evidence of high CO_2_ acclimation

Components of the chloroplast (stroma, envelop, thylakoid membrane) and photosystems are of interest to understand strain HCA adaptation to high CO_2_. These cell components showed a similar pattern of contigs up-regulated (8 to 14 log_2_FC) and down-regulated (-8 to -2 log_2_FC), the former with a higher extent of change (S1 Fig). Consequently, strain HCA can be correlated to a high flux of carbon since photosynthesis is the process that provides NADPH and ATP necessary to fix CO_2_ via the Calvin cycle [64]. In addition, Photosystem II has been reported to be highly sensitive to stress conditions; when *Chlorococcum littorale* and *Chlorella* sp. growing under 3% CO_2_ were transferred to low CO_2_ (0.04%) and extremely high CO_2_ (40%), the PI/PII ratio increased [65]. The over-regulation of the two photosystems in strain HCA suggests a higher tolerance to continuous supply of MFG, even under limited N provided by the NO_x_ component.

Despite N limitations, both strains grew under MFG with no difference in biomass productivity (Table 1). However, a higher initial growth rate was observed in the HCA strain, which could evidence a greater adaptation capability. During growth, N levels in solution for strain HCA remained less than 1 mg L^-1^ N compared to strain LCA (Fig 3f and 3g); this can be related to the 140 up-regulated contigs associated with N transporters that showed higher log_2_FC of 3 to 14 than the less abundant down-regulated contigs (-9 to -2 log_2_FC) (Fig 8a). Valenzuela *et al*. [61] also observed many up-regulated contigs for nitrate, ammonium, and urea transport under N depletion and suggested that it could represent a strategy to scavenge N from the medium. Following a higher expression of N transporters in strain HCA, nitrate reductase was also up-regulated.

### High CO_2_ acclimated microalgae present a lipidic profile enriched in glycerophospholipids during exponential growth under model flue gas

Lipidic composition in microalgae is known to be affected by growth conditions. Under optimal conditions, fatty acids are synthesized and used to generate membrane lipids such as glycerophospholipids (GP) [12, 66, 67], while under stress, glycerolipids (GL) tend to accumulate [8, 60]. Lipidome analysis showed that strain LCA presented higher GL intensities than HCA, which suggests a stressful condition under MFG (Fig 9c and 9d). These differences were more notorious at day 4 when strains were growing exponentially than during early stationary phase (day 5). In agreement, LCA growth rate at the beginning of the experiment was lower than strain HCA (Fig 3a and 3b), and it appears that N assimilation from MFG occurred later compared to HCA (Fig 3f and 3g).

In contrast, strain HCA presented higher intensities in most GP identified (Fig 9e and 9f). As mentioned above, GP is necessary for active growth, as observed in strain HCA from the beginning of the experimental period. The main features with significant changes matching GP m/z were structural lipids such as glycerophosphocholines (PC) and glycerophosphoethanolamines (PE). PC are found in mitochondria, endoplasmic reticulum, and plasmatic membranes as major GP [68, 69]. Similarly, PE are primarily found in the plasma membrane, which stabilizes the structure, protein integration, and cellular response [69, 70]. On the other hand, glycerophosphates (PA) act mainly as precursors of diacylglycerols (DG), glycerophosphoinositols (PI), and glycerophosphoserines (PS) [71–73]. PI are related to cell signaling under stress conditions [70, 73, 74]; however, PI response was similar between strains with two features higher in strain HCA and other two in LCA, probably evidencing differential response to the MFG condition (Fig 9c). Previous studies have found different behaviors on concentrations of PI when microalgae are under limited-N [70, 73, 75]. In agreement, in the present study, N supplied by the MFG was lower than the concentration of the complete culture medium, and microalgae strains seemed to respond differently to this condition. The general increase in GP in strain HCA correlated with the ability to use NO_x_ as a N-source from flue gas and probably a better strategy to scavenge N from limited concentrations, which did not appear to severely affect strain HCA as it affected strain LCA.

### Flue gas affects cell structure and biomass composition in a strain-dependent manner

Strain HCA exhibited a thicker cell wall that appears to be a consequence of years of acclimation to high CO_2_. In agreement, several studies have reported a higher content of cell wall components in microalgae under high CO_2_ [64, 76, 77]. Solovchenko *et al*. [77] described for *Desmodesmus* sp. that the cell wall polysaccharide layer acted as a sink for the excessive content of photosynthates under high CO_2_. Likewise, several *Chlorella* species accumulate uronic acid at high CO_2_, a polysaccharide precursor [76]. In addition, Cheng *et al*. [78] observed a thicker cell wall under conditions of limited N and P because protoplast and cell wall synthesis occurred faster than cell division. In our study, this response was also observed as an immediate adaptation to high CO_2_, and potentially also influenced by the limited N condition under MFG, when the low CO_2_ acclimation strain (LCA) was grown at 25% CO_2._ We observed a 1.7-fold increase in wall thickness from an air atmosphere to 25% CO_2_ in LCA (data not shown). However, cell wall thickness in strain LCA did not reach values observed in HCA (Fig 10d and 10h). Therefore, it appears that strain HCA has acquired a thicker cell wall because of years of acclimation to high CO_2_.

Considering that both strains can grow and use waste components of flue gas as a nutrient source, their conversion into valuable byproducts is of interest. However, differences between strains were observed in the composition of the biomass at the end of the experimental period (day 5). Pigment content was higher in strain HCA by 35% *Chl a*, 44% *Chl b*, and 32% carotenoids (Fig 10i), evidencing active photosynthetic activity and a higher rate of N intake being an essential element of the porphyrin ring in chlorophyll [61, 73]. Pancha *et al*. [79] reported that *Secenedesmus sp*. presented 75% less chlorophyll when nitrate was reduced from 247 to 0 mg L^-1^, as it has been reported for carotenoids. Lower concentrations of pigments have been interpreted as an indicator of stress under N-limited conditions in related species of the genus *Scenedesmus* [17, 61]. In our experimental condition, either caused by limited N under MFG or by using chlorophyll as a N-source. However, protein content that also requires N for their synthesis [49, 80], did not differ between strains (Fig 10j).

Conversely, strain LCA showed a higher content of starch and lipids compared to strain HCA (Fig 10j). This difference was also evident in TEM micrographs (Fig 10c and 10g) and validates transcriptome and lipidome data where it was suggested that more C was being directed to lipid synthesis of reserve molecules as glycerolipids, which were enriched in the LCA lipidome. We hypothesize that the condition of MFG with a limited supply of N represented a higher stress for strain LCA, and thus photosynthates were directed to the accumulation of storage compounds such as starch and lipids, as reported elsewhere [58].

## Conclusions

*D*. *abundans* strains HCA and LCA tolerated, grew, and used MFG components as a nutrient source. However, initial growth and N consumption from MFG was superior in strain HCA reaching maximum productivity a day before strain LCA. Annotation of *de novo* transcriptome evidenced the lack of information in microalgae genomics. Transcriptome changes in strain HCA compared to LCA were related to a higher internal carbon flux, the capacity to regulate intracellular acidification by using proton pumps, and a better strategy to scavenge N from MFG. As a result, all central carbon metabolism pathways, starch catabolism and anabolism, and TG biosynthesis presented higher up-regulated contigs and expression levels. Characteristic features of adaptation or tolerance to high CO_2_ might be related to proton pumps, N transporters, active synthesis of essential macromolecules for growth (nucleotides and amino acids), and cellular components of the photosynthetic apparatus. The strains’ N transporters might possess different substrate affinities or gene regulators, as in strain LCA, dissolved N accumulated to the third day of growth but not in HCA. Higher intensities of putative glycerophospholipids and lower intensities of glycerolipids in strain HCA evidence an active culture with fewer lipid reserves, as confirmed by the biochemical analysis. Overall, these results suggest that microalgae strains exhibit different levels of adaptability and response to MFG, depending on their history of cultivation. Understanding the molecular mechanisms underlying microalgae adaptation and response to MFG could contribute to develop improved strains and cultivation strategies for sustainable biotechnology applications.

## Supporting information

S1 Fig Expression level (log_2_FC) of contigs annotated as chloroplast (a) and photosystems (b) cell components GO terms. (DOCX)

S1 Table Transcriptome annotations. (XLSX)

S2 Table DEGs in KEGG pathways between strain HCA and LCA. (XLSX)

S3 Table Annotation results, DEGs log_2_FC and adjusted p.value, expression level, and RPKM of contigs encoded in central carbon metabolism. (XLSX)

S4 Table Annotation results, DEGs log_2_FC and adjusted p.value, expression level, and RPKM of contigs encoded one carbon, starch, and TAG metabolism. (XLSX)

S5 Table Annotation results, DEGs log_2_FC and adjusted p.value, expression level, and RPKM of contigs encoded in nitrogen metabolism. (XLSX)

S1 File RT-qPCR primers and optimization data. (DOCX)

S2 File KEGG pathways for purine, pyrimidine, and glycerophospholipids metabolism with annotated DEGs. (DOCX)
