# Supplementary material for: De novo transcriptome and lipidome analysis of Desmodesmus abundans under model flue gas reveals adaptive changes after ten years of acclimation to high CO2
Source: PLoS One. 2024 May 17;19(5):e0299780. doi: 10.1371/journal.pone.0299780 (PMC11101044; doi:10.1371/journal.pone.0299780)
Supplement: S1 Fig — (DOCX) [file pone.0299780.s001.docx]

**S1 Fig. Expression level (log_2_FC) of contigs annotated as chloroplast (a) and photosystems (b) cell components GO terms.**
