# Supplementary material for: De novo transcriptome and lipidome analysis of Desmodesmus abundans under model flue gas reveals adaptive changes after ten years of acclimation to high CO2
Source: PLoS One. 2024 May 17;19(5):e0299780. doi: 10.1371/journal.pone.0299780 (PMC11101044; doi:10.1371/journal.pone.0299780)
Supplement: S1 File — (DOCX) [file pone.0299780.s007.docx]

**Table S1.** RT-qPCR primers and optimization data.

| **Gene** | **Size (bp)** | **Primer sequences** | | **Calibration curve linear correlation R^2^** | **Optimized cDNA concentration (ng)** | **Optimized primer concentration (nM)** | |
| --- | --- | --- | --- | --- | --- | --- | --- |
|  |  | **Forward (5´- 3´)** | **Reverse (5´- 3´)** |  |  | **Forward** | **Reverse** |
| Ammonium transporter (AMT) | 136 | GCTGTTGAACCCAAACCAGC | GATTGGTGCCAAGTTCTGTGG | 0.975 | 5 | 300 | 300 |
| Ammonium transporter (AMT3) | 145 | AGCCKGCAAAGTCRATCATG | AACGCYTACRTTGCCTACAC | 0.976 | 5 | 200 | 300 |
| Urea transporter (DUR) | 93 | CAGCAGGTAKCCCTTRTASG | GGRCTSGTSTTTGGRATCATC | 0.995 | 5 | 300 | 300 |
| Nitrate transporter (NTR1) | 95 | ACGCAGCAGGCTTCATCATC | GGTGTTGAACATGCTGGTGC | 0.976 | 5 | 200 | 300 |
| Nitrate transporter (NTR2) | 123 | CCATCTTCGGCTGCATGAAC | CTCCCAGGGTCTGGATAATCC | 0.989 | 5 | 200 | 300 |
| Housekeeping gene (18S rDNA) | 188 | GGACCTGCTTCTGGGCTTCA | TACGAATGCCCCCGACTGTT | 0.990 | 0.05 | 300 | 200 |
