# Supplementary material for: De novo transcriptome and lipidome analysis of Desmodesmus abundans under model flue gas reveals adaptive changes after ten years of acclimation to high CO2
Source: PLoS One. 2024 May 17;19(5):e0299780. doi: 10.1371/journal.pone.0299780 (PMC11101044; doi:10.1371/journal.pone.0299780)

**S2 File. KEGG pathways for purine (a), pyrimidine (b), and glycerophospholipids (c) metabolism with annotated DEGs.** Enzymes in color correspond to DEGs.

**a)**
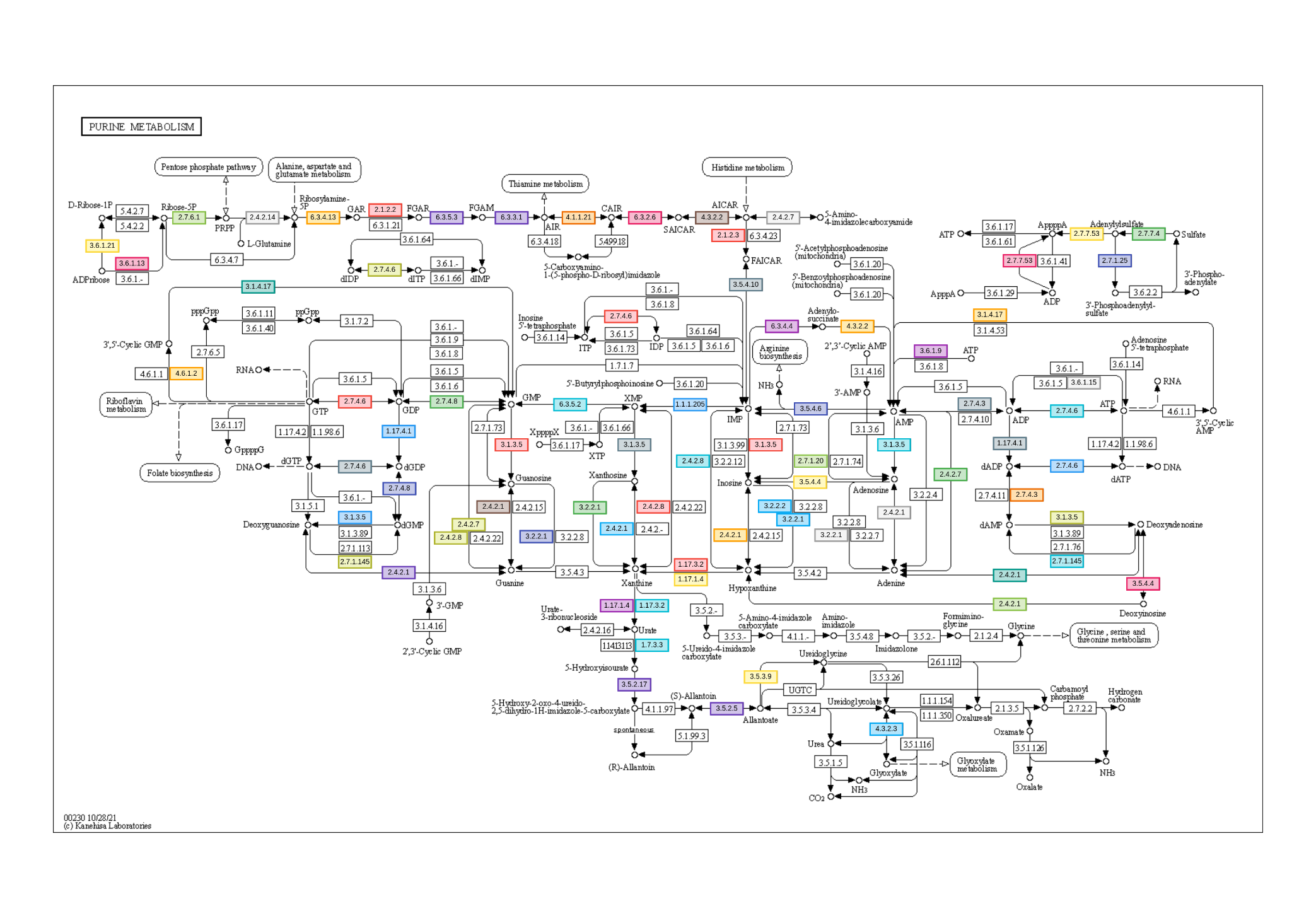


**b)**


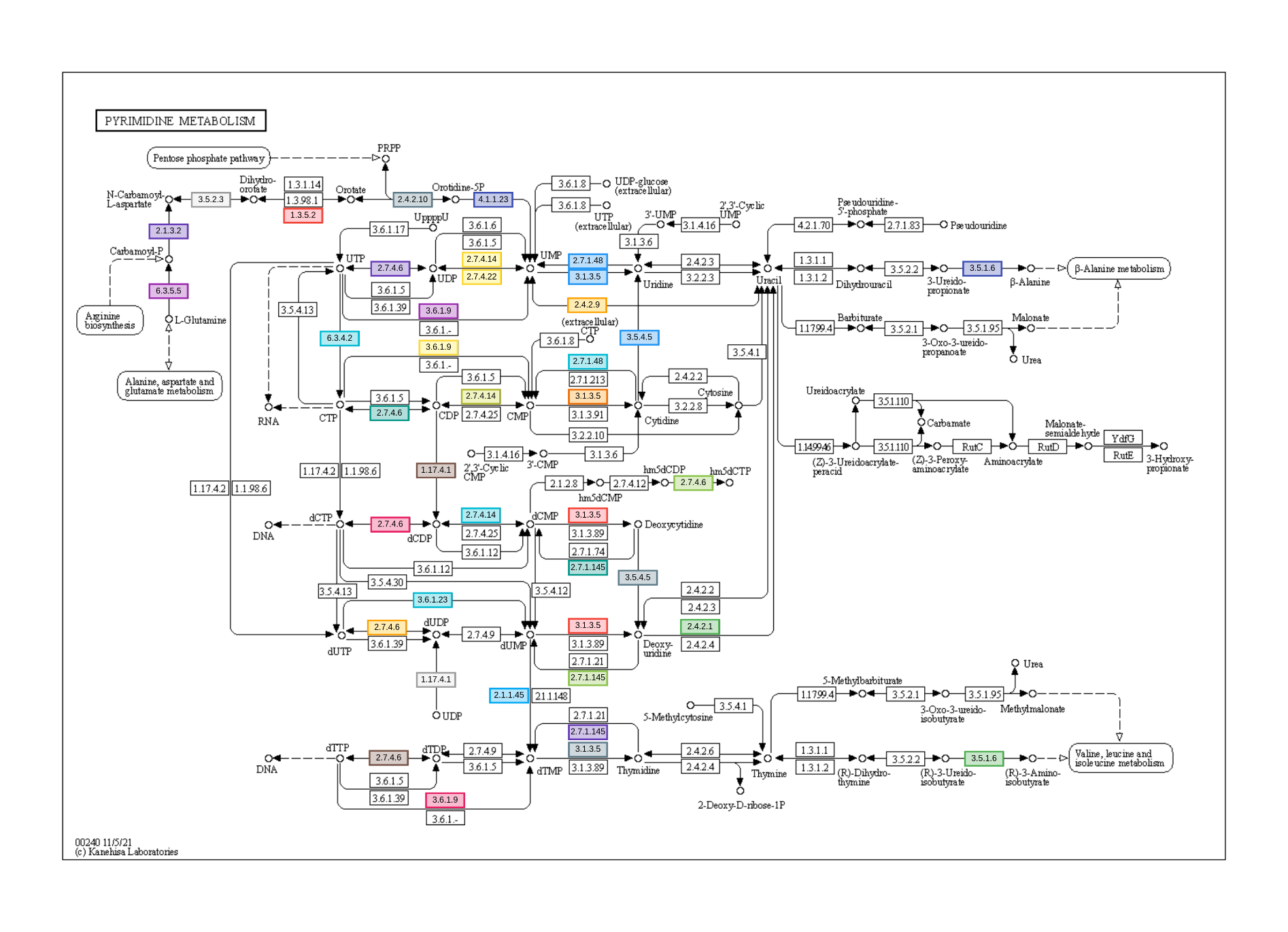


**c)**


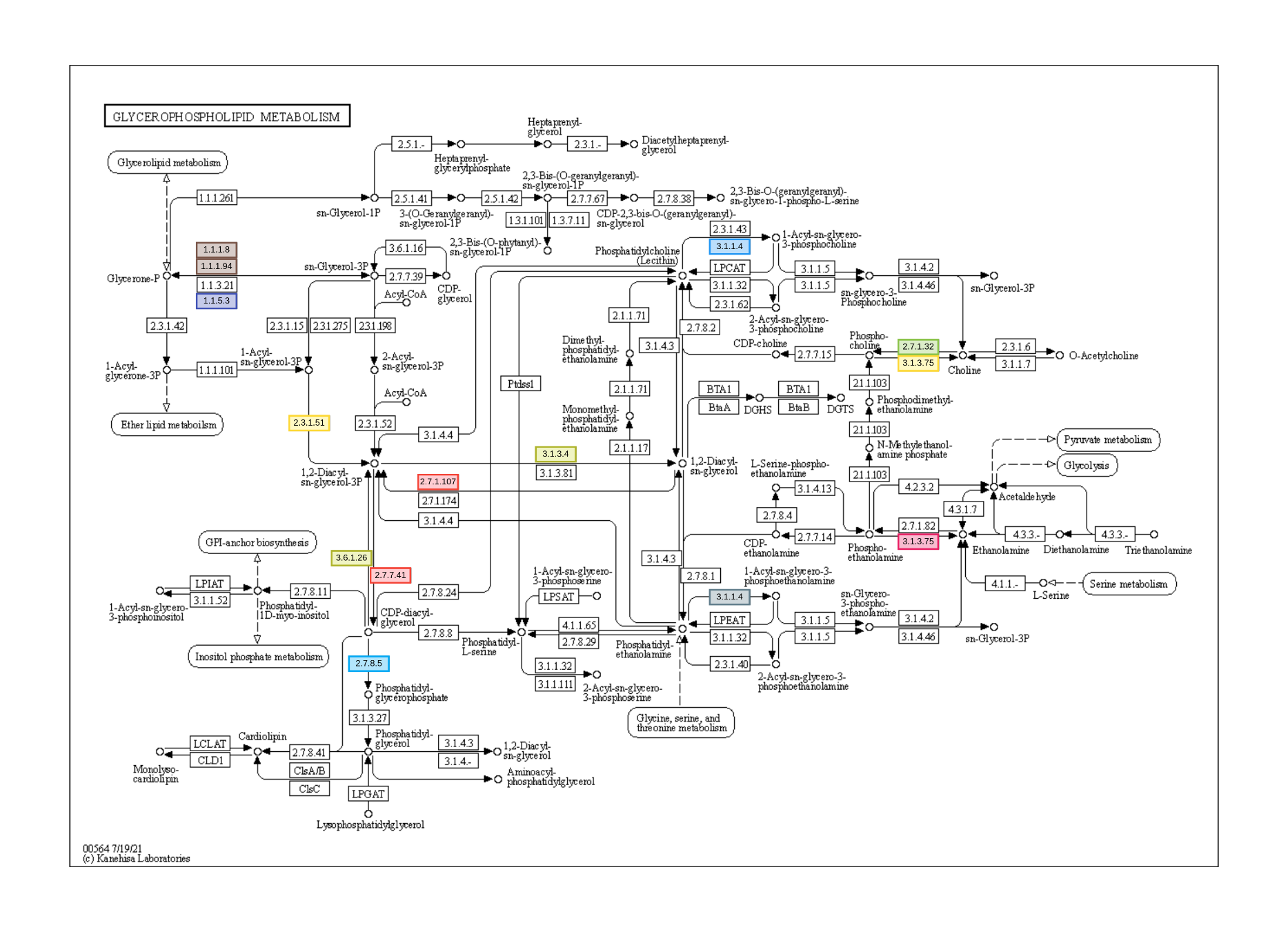

Supplement: S2 File — (DOCX) [file pone.0299780.s008.docx]
